# Canonical transcription termination mechanisms explain a minority of operons in cyanobacteria

**DOI:** 10.1128/msystems.01581-25

**Published:** 2026-05-18

**Authors:** Jennifer A. Cascino, K. Julia Dierksheide, Rishi K. Vishwakarma, Yulia Yuzenkova, Paul Babitzke, Katsuhiko S. Murakami, Gene-Wei Li

**Affiliations:** 1 Department of Biology, Massachusetts Institute of Technology 311383, Cambridge, Massachusetts, USA; 2 Department of Biochemistry and Molecular Biology, Center for RNA Molecular Biology, Pennsylvania State University 189499, University Park, Pennsylvania, USA; 3 Department of Structural Biology, St. Jude Children’s Research Hospital 467026, Memphis, Tennessee, USA; 4 Centre for Bacterial Cell Biology, Faculty of Medical Sciences, Biosciences Institute, Newcastle University 12186, Newcastle upon Tyne, United Kingdom; 5 Center for Structural Biology, Huck Institutes of the Life Sciences, Pennsylvania State University 124474, University Park, Pennsylvania, USA; 6 Howard Hughes Medical Institute, Cambridge, Massachusetts, USA; University of Memphis, Memphis, Tennessee, USA

**Keywords:** cyanobacteria, transcription termination, transcription, transcriptional regulation, picocyanobacteria, gene regulation, transcriptomics, intrinsic termination, Mfd, RNA sequencing

## Abstract

Cyanobacteria are abundant phototrophs and hold potential as a carbon-negative platform for bioengineering applications. However, these efforts have been hampered by limited mechanistic understanding of their gene expression, including transcription termination. Unlike most bacteria, cyanobacteria lack the transcription termination factor Rho, raising the speculation that all transcription ends with intrinsic terminators. Here, we show that most transcription units (TUs) in *Synechococcus elongatus* PCC 7942 are not terminated by known termination pathways. Although many TUs (52%) have unique, well-defined 3′ ends, only a small fraction have features that resemble canonical intrinsic terminators (22%). The noncanonical 3′ ends broadly lacked strong secondary structure, making it unclear how these ends are protected against 3′−5′ exonucleolytic decay. Furthermore, many TUs (46%) have diverse positions of mRNA 3′ ends, suggesting a potentially diffuse termination signal. Finally, we observed a moderate increase in RNA levels downstream of most defined 3′ ends in the absence of the transcription-repair coupling factor Mfd. This finding indicates that Mfd plays a limited, but widespread, role in RNA end formation, potentially through termination of stalled RNAPs. Together, our work reveals unique end architectures of the cyanobacterial transcriptome and suggests that undescribed transcription termination mechanisms are active in the phylum.

**IMPORTANCE:**

Our understanding of bacterial transcription regulation is largely based on model organisms like *Escherichia coli* and *Bacillus subtilis*, yet many of these mechanisms appear absent or divergent in cyanobacteria. These differences limit our fundamental understanding of gene regulation and the applied potential of cyanobacteria in sustainable biomanufacturing. To address this gap, we characterized transcription termination in the model cyanobacterium *Synechococcus elongatus* PCC 7942. We resolve a longstanding question by showing that intrinsic termination alone cannot account for most termination events in this organism. Pervasive transcript ends lacking intrinsic terminator features and the absence of Rho suggest the existence of novel termination mechanism(s) and highlight a largely unexplored regulatory landscape. Simultaneously, our work expands the repertoire of functionally characterized cyanobacterial intrinsic terminators, offering a new toolkit to fine-tune gene expression using terminators of defined strengths. These findings pave the way for more predictable and powerful applications of cyanobacteria in green biotechnology.

## INTRODUCTION

Transcription termination is a critical regulatory step in gene expression for generating accurate RNA products and recycling the RNA polymerase (RNAP) (1, 2). The point of termination shapes the 3′ untranslated region and mRNA stability, while the efficiency of termination determines levels of downstream gene expression (3–8). Furthermore, failure to terminate RNAP can have harmful effects on cells, including decreased production of functional transcripts and increases in antisense transcription or transcription-replication conflicts (1, 2, 9, 10). Despite this crucial status, the mechanisms for terminating transcription in cyanobacteria—prominent contributors to global carbon fixation (11)—remain elusive.

In most bacteria, two major termination mechanisms are recognized (1, 2). Named for their dependence on the trans-acting termination factor Rho (12), these pathways are known as Rho-dependent and Rho-independent (or intrinsic) termination. In Rho-dependent termination, the homohexamer Rho binds the nascent transcript at pyrimidine-rich, unstructured regions called Rho-utilization (*rut*) sites (1, 2). The helicase and ATPase activities of Rho dislodge the RNA, collapsing the transcription elongation complex (EC) (1, 2). Intrinsic termination instead relies on two requisite sequence motifs in the RNA—a GC-rich hairpin followed by a uridine-rich region (U-tract) (1, 2, 13–15). These features destabilize ECs by pausing RNAP at the U-tract, allowing hairpin folding to trigger EC collapse (1, 2, 13–15). Many *in silico* platforms have been designed to predict intrinsic terminators from bacterial genomes (16–33). High-throughput sequencing of bacterial transcriptomes has also been used for genome-wide identification of intrinsic terminators using RNA 3′ ends (5, 8, 34–47).

Contrary to most bacteria, cyanobacteria do not encode a known homolog of Rho (48–51) and have poorly characterized intrinsic terminators, leaving it broadly unclear how they terminate transcription. Given Rho’s absence, it is commonly proposed that termination occurs only through the intrinsic mechanism in cyanobacteria (49, 52–56), but this hypothesis has not been tested systematically. Previous studies using RNA sequencing or genomic data showed a varying degree of prevalence for intrinsic terminators, but the definition of intrinsic terminator used in these studies did not always include both a hairpin and a U-tract, which are considered the classic features (41, 49, 52, 57). To date, only nine putative cyanobacterial intrinsic terminators have been tested in their native context, where they exhibited variable termination efficiencies and often performed worse than *E. coli*-derived or synthetic terminators (48, 55, 56). Taken together, the prevalence of intrinsic terminators in the cyanobacterial transcriptome remains unclear.

Understanding transcription termination in cyanobacteria is critical given their significance to global photosynthesis and, increasingly, biomanufacturing. Cyanobacteria are the only bacteria capable of oxygenic photosynthesis and are estimated to perform at least 20%–30% of global carbon fixation (58, 59). As such, they have been hailed as potential platforms for industrial-scale, sustainable chemical synthesis that takes advantage of their unique ability to fix atmospheric CO_2_ into other carbon-containing metabolites using water and light (59–66). However, metabolic engineering of cyanobacteria is hindered by poor characterization of genetic parts like transcription terminators (48, 66).

Accurate mapping of RNA 3′ ends can be used to determine the prevalence of bacterial termination mechanisms. In *E. coli*, for example, high-resolution RNA 3′ end mapping showed that ~40% of precisely defined transcript termini correspond to intrinsic terminators, while another large portion (~30%) are formed by Rho-dependent termination (5). Transcripts insensitive to the Rho inhibitor bicyclomycin had a GC-rich hairpin followed by a U-tract upstream of the 3′ end, consistent with intrinsic terminators. By contrast, bicyclomycin-sensitive transcripts had GC-rich hairpins without a strong U-tract at the 3′ end. These transcripts were interpreted to undergo Rho-dependent termination at diffuse positions downstream, followed by exonucleolytic processing to a stabilizing hairpin at the measured end (5). Consistent with this model, the sequence downstream of these ends was C-rich and G-poor, resembling a *rut* site signature (1, 2, 5). By determining the precise end position of transcripts, high-resolution RNA 3′ end mapping in combination with sequence analysis can thus help reveal the termination mechanism underlying their formation and assess intrinsic terminator prevalence in cyanobacteria.

In this study, we used end-enriched RNA sequencing (Rend-seq) (8) to map RNA 3′ end architectures at single-nucleotide resolution across hundreds of operons in the freshwater cyanobacterium *Synechococcus elongatus* PCC 7942 (*Syn*). With this proverbial magnifying lens on RNA 3′ ends, we classified the *Syn* transcriptome into two types of transcription units (TUs): those with clearly defined 3′ ends and those with diffuse, multi-position 3′ ends. We found that most TUs in *Syn* (52%) have defined 3′ ends, but diffuse ends are also prevalent (46%). About 43% of TUs with defined ends—only 22% of all TUs—have signatures of intrinsic terminators (a hairpin and U-tract), indicating this termination mechanism is far less prevalent than previously thought. The defined ends lacking intrinsic terminator features do not show strong secondary structure but exhibit a 5′-TG-3′ dinucleotide motif that partially overlaps with the bacterial elemental pause sequence, which encodes a signal for RNAP to isomerize into a transiently paused state (67). The diffuse ends do not have nearby identifiable intrinsic terminators or other shared sequence features. Finally, we examined the role of other potential termination factors. Interestingly, deleting the bacterial transcription-repair coupling factor Mfd increased apparent transcriptional readthrough at most intrinsic terminators and other defined ends without shifting the position of termination, which may be explained by a role in enhancing termination at or downstream of the observed RNA 3′ ends. Together, our work reveals that intrinsic termination plays a minor role in *Syn*, identifies a potential contribution from Mfd, and suggests that undescribed transcription termination mechanisms must operate in the phylum to explain how most operons are punctuated.

## RESULTS

### Mapping RNA 3′ ends reveals defined and diffuse end classes

Rend-seq can simultaneously detect the positions of transcription unit (TU) 5′ and 3′ ends at single-nucleotide (nt) resolution, enabling the precise definition of TU boundaries (8). To assess the prevalence of intrinsic termination in *Syn*, we measured RNA 3′ end positions in the transcriptome of exponential-phase cells grown in BG-11 on a 12 h:12 h light:dark diel cycle using Rend-seq and characterized the sequence features associated with those ends.

Transcript 3′ ends typically exhibit two main profiles. Some TUs end at a sharply-defined position—concentrated at one or a few bases—producing “defined ends.” Others lack a dominant endpoint and instead display a broad distribution of 3′ ends across many positions, resulting in “diffuse ends.” These profiles reflect the diversity of RNA molecules expressed from a gene and can provide insight into the termination mechanisms that generated them. In *E. coli*, for instance, defined ends can result from either intrinsic termination, where the terminator produces RNA molecules of uniform length, or Rho-dependent termination in which diffusely terminated RNAs are trimmed by exoribonucleases to a stabilizing hairpin (5). By contrast, the origins of diffuse TU ends are less well understood. Possible explanations include Rho-dependent (or other factor-dependent) termination without trimming, incomplete exoribonucleolytic decay, or uncharacterized termination mechanisms that do not rely on a single dominant site. Thus, TU 3′ end profiles can inform both the diversity of transcript isoforms and the termination pathway underlying them.

**Fig 1:**
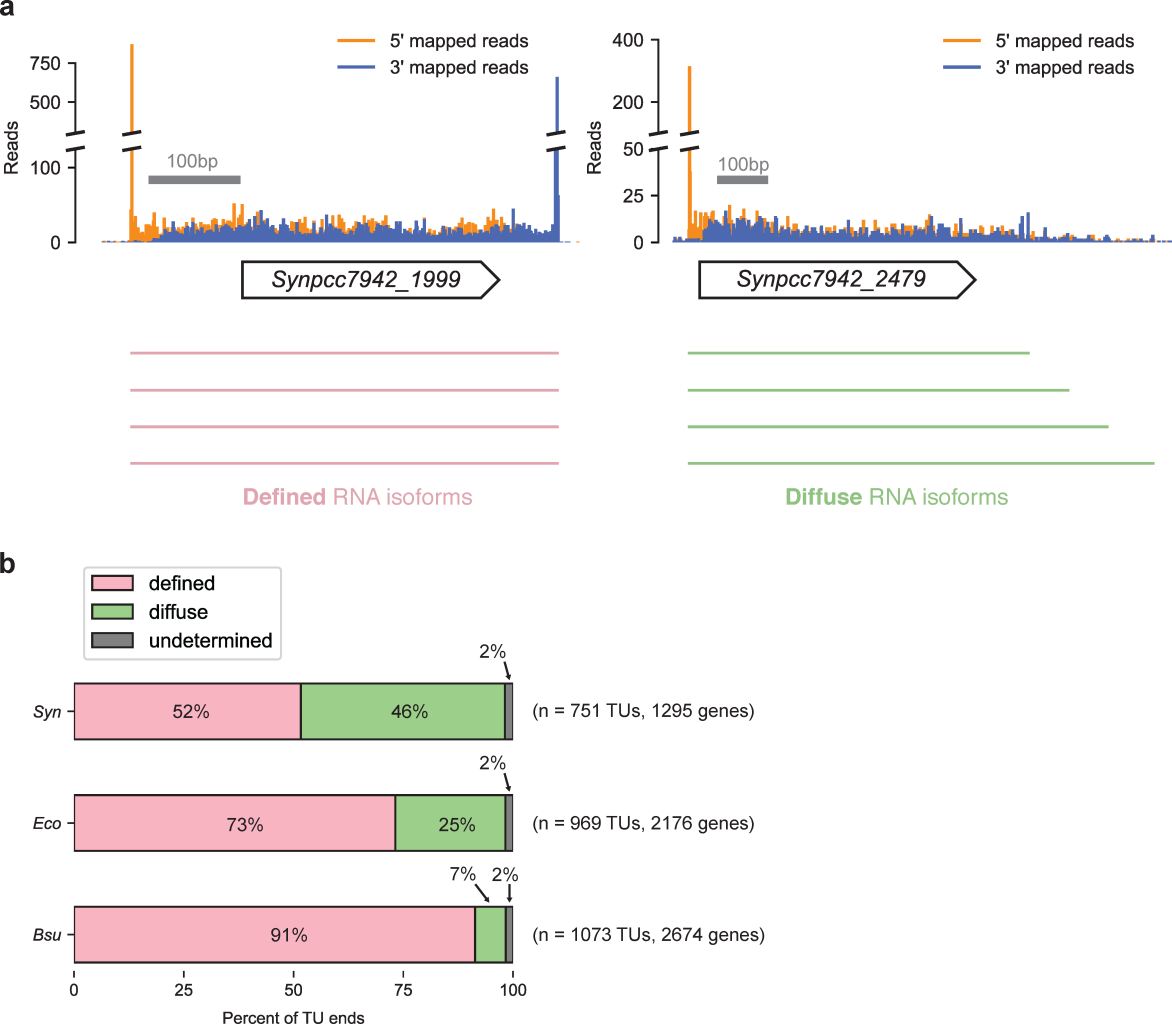
Comparative analysis of TU 3′ end profiles using Rend-seq. (**a**) Representative Rend-seq traces illustrating defined versus diffuse TU 3′ end profiles. Defined ends exhibit a sharp 3′ peak followed by immediate signal drop-off, corresponding to RNA molecules of uniform length (left), while diffuse ends show a gradual decrease in 3′-mapped reads and lack a dominant end position, corresponding to heterogeneous RNA isoforms (right). Gray bar shows *x*-axis scale. (**b**) Genome-wide classification of TU 3′ ends in *Synechococcus elongatus* PCC 7942 (*Syn*), *E. coli* MG1655 (*Eco*), and *B. subtilis* 168 (*Bsu*) using an automated end scoring pipeline (Supplemental Methods). TUs were classified as having either defined (pink) or diffuse (green) 3′ ends based on parameters in Rend-seq data. End types at a minor subset of TUs could not be determined (gray). Only TUs with sufficient Rend-seq coverage were included. Total number of genes analyzed and corresponding number of TUs (excluding genes internal to TUs) are shown to the right of the bars.

Importantly, Rend-seq can distinguish defined and diffuse TU end profiles. In these data, TUs with defined ends exhibit a sharp peak in 3′-mapped reads (3′ peak) followed by an immediate signal drop-off after the peak (Fig. 1a; see Supplemental Methods for Rend-seq peak definition). By contrast, TUs with diffuse ends show a gradual decrease in signal in the 3′-mapped channel (Fig. 1a) or, in some cases, several 3′ peaks (Supplemental Methods). To automatically classify the 3′ ends of *Syn* TUs as defined or diffuse, we developed a computational pipeline that scores end profiles based on Rend-seq data (Supplemental Methods). As a reference for interpreting the *Syn* data, we applied the same pipeline to previously published Rend-seq data sets from *E. coli* MG1655 (*Eco*) and *B. subtilis* 168 (*Bsu*) (8)—well-characterized, Rho-encoding model organisms.

Application of the automatic end scoring pipeline revealed that only a slight majority of *Syn* TUs have defined ends. Of the 751 TUs (1,295 genes) with sufficient sequencing coverage, 52% (*n* = 388) had defined 3′ ends (Fig. 1b; Table S1). Remarkably, a substantial portion of TUs (46%, *n* = 349) had diffuse ends lacking a well-defined 3′ end position. This finding contrasts with the Rho-encoding species, where the vast majority of TUs have defined ends. Indeed, in *Eco* and *Bsu*, defined ends account for 73% (*n* = 709/969) and 91% (*n* = 980/1,073) of TUs, respectively (Fig. 1b; Table S1). These comparisons are based on similar genome coverage, with the pipeline scoring about half of the annotated genes in *Syn* (*n* = 1,295/2,714), *Eco* (*n* = 2,176/4,651), and *Bsu* (*n* = 2,674/4,546). Thus, in *Syn*, defined 3′ ends account for roughly half of TU ends, whereas diffuse ends are markedly more prevalent than in Rho-encoding bacteria like *Eco* and *Bsu*, suggesting a possible point of divergence in termination dynamics.

### Minority of TU ends are consistent with intrinsic termination

While defined 3′ ends can be produced by both canonical termination pathways, only transcripts punctuated by intrinsic terminators are expected to have a hairpin and a U-tract immediately upstream of the defined end (5). To assess the prevalence of intrinsic termination in *Syn*, we identified the subset of defined ends with both features.

**Fig 2:**
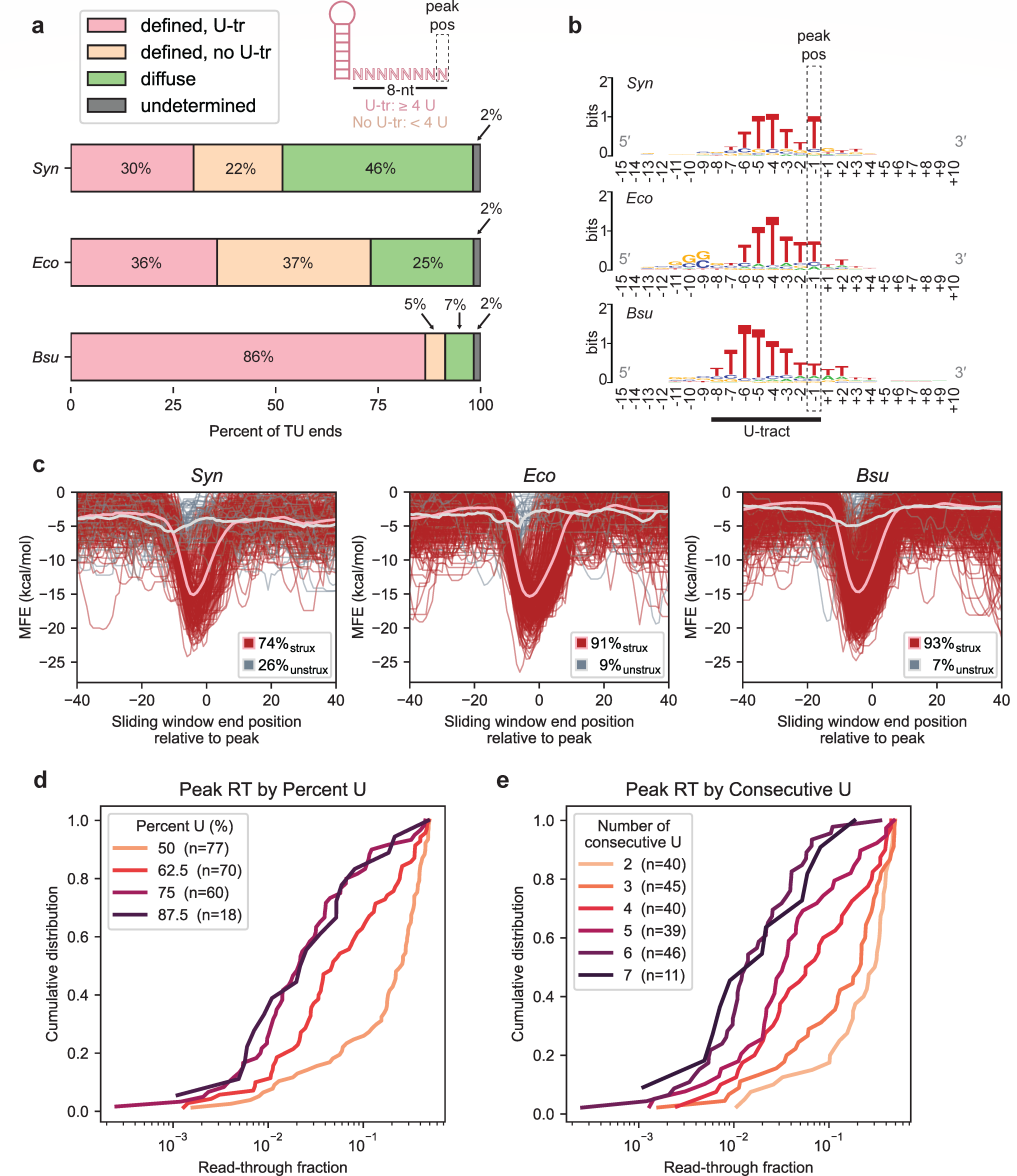
Defined TU 3′ ends with U-tracts are consistent with intrinsic termination. (**a**) Proportion of defined TU 3′ ends with (pink) or without (light orange) putative U-tracts in *Synechococcus elongatus* PCC 7942 (*Syn*), *E. coli* MG1655 (*Eco*), and *B. subtilis* 168 (*Bsu*). U-tracts are defined as ≥ 4 uridines in the 8 nucleotides (nt) directly preceding and including the 3′ peak, which defines the end position. (**b**) Sequence logos for 25-nt sequence context at defined ends with U-tracts. Peak position and putative U-tract region are shown. (**c**) Predicted RNA secondary structure at U-tract defined ends. For each end, minimum free energies (MFEs) of 30-nt sliding windows were computed for the 110-nt sequence context of the 3′ peak (Supplemental Methods). Traces with an MFE < −10 kcal/mol for any of the windows ending from 10 nt upstream of the peak (−10 on *x*-axis) to the peak itself (0 on *x*-axis) were colored red to convey the frequency of ends with high RNA secondary structure in the region upstream of the peak where a terminator hairpin is expected to fold. Traces that did not meet this criterion were colored gray to convey the frequency of ends that are not highly structured in the terminator hairpin region. Overlaid pink and light gray traces represent the average MFE trace for ends classified as highly structured (red traces) and not highly structured (gray traces), respectively. Frequency of structured and unstructured ends are listed in inset. (**d**) Cumulative distribution of readthrough levels at *Syn* U-tract defined ends as a function of U-content (%U) in the putative U-tract region. Number of ends for each category are listed. (**e**) Cumulative distribution of readthrough levels at *Syn* U-tract defined ends as a function of U-tract length (maximum number of consecutive uridines) in the putative U-tract region. Number of ends for each category are listed. Ends with no consecutive uridines (*N* = 4) have been omitted. For panels d and e, readthrough was calculated as described in Supplemental Methods. Peak pos = 3′ peak (defined end) position; U-tr = U-tract; strux = structured; unstrux = unstructured; RT = transcriptional readthrough.

We found that 58% (*n* = 225) of *Syn* defined ends have ≥4 U in the 8 nt upstream of the 3′ peak, representing 30% of all *Syn* TUs (Fig. 2a; Table S2) (19% of defined ends have 3 U in this region, similar to the probability of 17% by random chance [Fig. S1]). By comparison, many more putative U-tracts (≥4 U in 8-nt upstream window) were found in *Bsu* (95% of defined ends, representing 86% of all TUs, *n* = 928) (Fig. 2a), consistent with reports that intrinsic termination predominates in Bacillota like *Bsu*, where Rho is dispensable and predicted to play a minor role in terminating coding transcription (22). We uncovered a similar number of putative U-tracts in *Eco* (49% of defined ends, representing 36% of all TUs, *n* = 346) as in *Syn* (Fig. 2a), aligning with evidence that Rho-dependent termination plays a major role in *Eco*, where Rho is essential (22) and estimated to terminate 20%–50% of genes (5). Together, these data suggest intrinsic terminators are relatively rare in *Syn*, more closely resembling the termination landscape of *Eco*, which relies heavily on Rho, than that of *Bsu*, where intrinsic termination dominates.

Analysis of nucleotide information content around U-tract-containing defined ends revealed similar U-rich signatures in *Syn* and *Eco* (Fig. 2b). *Bsu* exhibited a more extended U-rich signal in the putative U-tract region (Fig. 2b), which suggests that, in addition to their greater prevalence, intrinsic terminators in *Bsu* have more complete U-tracts. In *Bsu*, and to a lesser extent *Eco* and *Syn*, the U-rich signature also extends slightly beyond the defined end. This could reflect how defined end positions were assigned, where closely spaced 3′ peaks (within ±5 nt) are collapsed to the single highest peak (by *z*-score, Supplemental Methods). These suppressed minor peaks could represent valid RNA isoforms produced by limited multi-position intrinsic termination. Alternatively, 3′−5′ exoribonucleolytic trimming could shift the measured peak into the U-tract. Notably, although the nucleotides at the 3′ end can be more variable than at other positions of the U-tract in *E. coli* (2), the U at the −1 (peak) position is strongly enriched in *Syn* (Fig. 2b).

We also confirmed that defined ends with U-tracts have strong secondary structure consistent with intrinsic terminator hairpins. Folding 30-nt sliding windows around the 3′ peak at these U-tract-containing ends *in silico* revealed widespread upstream structure in *Syn*, with 74% (*n* = 167/225) having a minimum free energy (MFE) below −10 kcal/mol in the expected intrinsic terminator hairpin region (Fig. 2c; Supplemental Methods). Most *Eco* and *Bsu* U-tract defined ends (91%–93%) also had strong secondary structure. Taken together, these analyses suggest that most defined ends with U-tracts in *Syn* are consistent with canonical intrinsic terminators, although defined ends with evidence of both an upstream hairpin and U-tract account for only 22% of all TUs (74% of 30% U-tract-containing defined ends).

To further demonstrate that U-tract defined ends in *Syn* are likely formed by intrinsic termination, we analyzed termination activity at these sites. Since stronger U-tracts are correlated with higher termination efficiency (i.e., reduced transcriptional readthrough) in *Bsu* intrinsic termination (8), we analyzed the relationship between U-tract composition and readthrough for all 225 *Syn* defined ends with U-tracts (Supplemental Methods). Ends with higher U-content or more consecutive uridines showed systematically lower readthrough, consistent with stronger termination (Fig. 2d and e). Thus, U-tract strength correlates with termination efficiency at *Syn* U-tract defined ends, supporting their function as intrinsic terminators. Finally, we used an *in vitro* transcription system to directly test the termination activity of the putative intrinsic terminators corresponding to two of the U-tract defined ends (Supplemental Methods). Both sequences terminated transcription of *Syn* RNAP and were stimulated by NusA, as seen for many intrinsic terminators in *B. subtilis* and *E. coli* (35, 68–72) (Fig. S2). In sum, these data indicate that only 22% of TUs in *Syn* are intrinsically terminated. Contrary to the prevailing model, canonical intrinsic termination does not appear to be the sole termination mechanism active in all cyanobacteria.

### Many defined 3′ ends are inconsistent with intrinsic termination

A fifth of all *Syn* TUs (22%, *n* = 163) have defined ends without U-tracts (Fig. 2a; Table S2). To investigate their origin, we profiled their sequence and structure features and compared them to known termination mechanisms. We also drew a comparison to defined ends lacking U-tracts found for over a third of *Eco* TUs (37%, *n* = 363) and a small fraction of *Bsu* TUs (5%, *n* = 52) (Fig. 2a).

**Fig 3:**
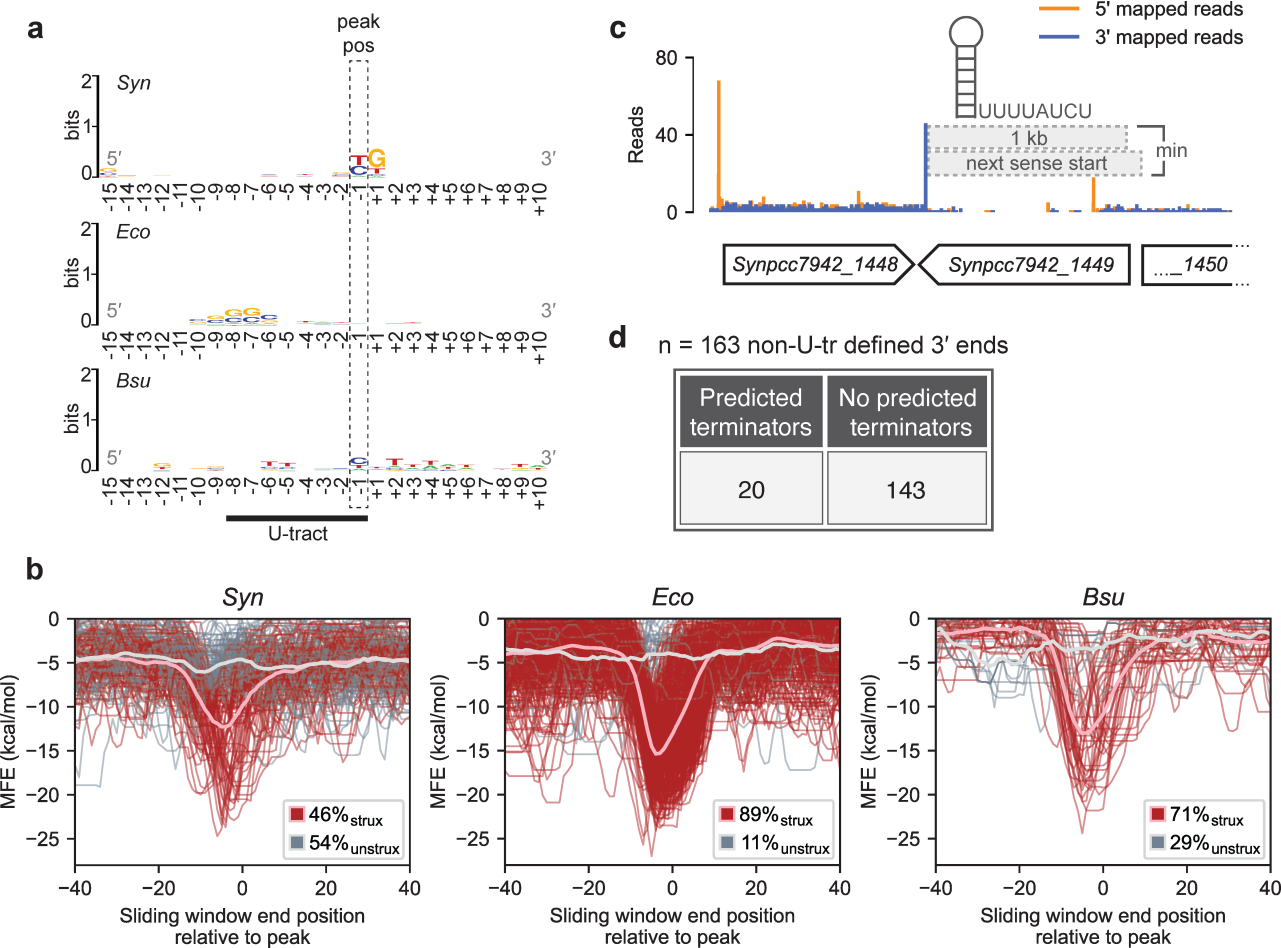
Defined TU 3′ ends without U-tracts are inconsistent with intrinsic termination. (**a**) Sequence logos for 25-nucleotide (nt) sequence context at defined ends without U-tracts (<4 uridines in 8 nt directly preceding and including the 3′ peak). Peak position and putative U-tract region are shown. (**b**) Predicted RNA secondary structure at non-U-tract defined ends. For each end, minimum free energies (MFEs) of 30-nt sliding windows were computed for the 110-nt sequence context of the 3′ peak (Supplemental Methods). Traces with an MFE < −10 kcal/mol for any of the windows ending from 10 nt upstream of the peak (−10 on *x*-axis) to the peak itself (0 on *x*-axis) were colored red to convey the frequency of ends with high RNA secondary structure in the region upstream of the peak where a terminator hairpin is expected to fold. Traces that did not meet this criterion were colored gray to convey the frequency of ends that are not highly structured in the terminator hairpin region. Overlaid pink and light gray traces represent the average MFE trace for ends classified as highly structured (red traces) and not highly structured (gray traces), respectively. Frequency of structured and unstructured ends is listed in inset. (**c**) Analysis of undetected intrinsic termination at non-U-tract defined ends. TransTermHP-predicted intrinsic terminators in the downstream intergenic region of defined ends lacking U-tracts were identified. Only terminators that passed a high confidence filter were included in the analysis (Supplemental Methods). The intergenic region is defined as the sequence from the stop codon of the gene under consideration to the next start codon on the same strand (limited to 1 kilobase if longer). One kilobase gray box is drawn to scale. (**d**) Frequency of non-U-tract defined ends with a high-confidence intrinsic terminator predicted in the downstream intergenic region. Peak pos = 3′ peak (defined end) position; *Syn* = *Synechococcus elongatus* PCC 7942; *Eco* = *E. coli* MG1655; *Bsu* = *B. subtilis* 168; strux = structured; unstrux = unstructured; *…_1450* = *Synpcc7942_1450*; kb = kilobase; min = minimum; U-tr = U-tract.

First, we determined that defined 3′ ends without apparent U-tracts are not formed by intrinsic terminators immediately downstream, followed by limited 3′−5′ exonucleolytic trimming that removes several nucleotides (i.e., part or all) of the U-tract. Sequence logos of the sequences surrounding these ends revealed no additional uridines downstream of the peak position (Fig. 3a). Furthermore, these ends in *Syn* also broadly lack the secondary structure associated with intrinsic termination, with only 46% (*n* = 75/163) showing strong upstream structure (Fig. 3b). By contrast, most *Eco* ends (89%, *n* = 324/363) exhibited high levels of structure, consistent with known roles of repetitive extragenic palindromic (REP) elements—GC-rich hairpins that stabilize RNA 3′ ends but do not mediate termination (73, 74). We also observed a motif matching the consensus REP element (73, 75) upstream of the peak position in *Eco* (Fig. 3a). These structures similarly align with prior observations that Rho-dependent termination followed by exonucleolytic processing to a stabilizing hairpin produces defined TU ends with GC-rich hairpins lacking U-tracts (5). In *Syn*, however, such stabilizing structures are largely absent. How the majority of these TU ends are formed and stabilized against exonucleolytic decay by PNPase or RNase II remains unclear, but local RNA secondary structure does not appear to be the predominant mechanism.

Interestingly, we observed a weak sequence motif at *Syn* defined ends lacking U-tracts—a 5′-TG-3′ dinucleotide at the peak and first downstream base (Fig. 3a). Overall, the dinucleotide appears at 34% of defined ends without U-tracts (*n* = 56/163). The origin of this motif is unknown, but multiple lines of evidence support its identification as an *in vivo* signature rather than a technical artifact like RNase contamination during library preparation. First, these RNA 3′ ends lack the corresponding downstream 5′ ends that should be formed upon RNase cleavage *in vitro*. Second, 5′-TG-3′ dinucleotide motifs in analyzed *Syn* genes rarely give rise to 3′ peaks—only 0.09% of such instances coincide with a peak (*n* = 80/88,426).

Finally, we assessed if defined ends lacking U-tracts might result from intrinsic termination events farther downstream, followed by extensive RNA processing. Although intrinsic terminator hairpins are considered to protect against exonucleolytic decay, endoribonucleases could separate the terminator from the transcript. Using a program to predict intrinsic terminators (TransTermHP) (23), we predicted putative intrinsic terminators in the *Syn* genome and quantified their presence downstream of genes in each end category (Fig. 3c and d; Supplemental Methods). For genes followed by defined ends lacking U-tracts, only 12% (*n* = 20/163) had a high-confidence intrinsic terminator in the downstream intergenic region, compared to ~59% of genes followed by defined ends with U-tracts (*n* = 132/225). This markedly lower frequency suggests that non-U-tract defined ends are unlikely to arise from undetected intrinsic termination.

The above sequence and structure analyses establish that defined ends without U-tracts in *Syn* are broadly inconsistent with intrinsic termination. These ends lack U-tracts at or immediately downstream of the peak position, and over half are not highly structured. Moreover, high-confidence intrinsic terminators are largely absent downstream. We observe a 5′-TG-3′ dinucleotide motif at the peak and +1 position, with evidence supporting its biological origin. However, its role in TU end formation remains unclear, and the broader mechanism(s) that terminate and stabilize these transcripts against decay are not known.

### RNA levels can ramp down long after genes without known terminators

**Fig 4:**
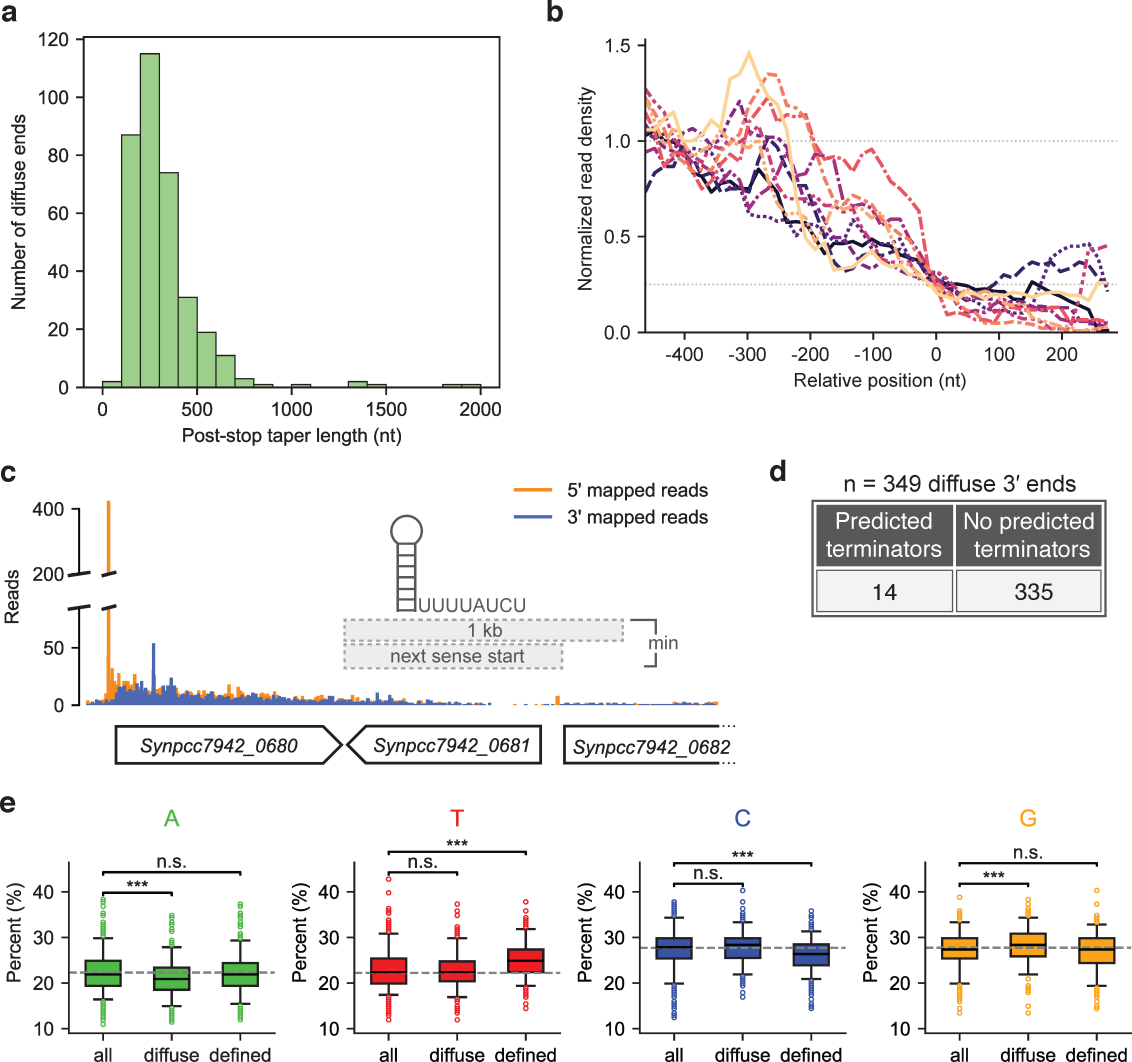
*Syn* diffuse TU 3′ ends taper long after genes without known terminators. (**a**) Distribution of post-stop tapering lengths for *Synechococcus elongatus* PCC 7942 (*Syn*) diffuse 3′ ends. Diffuse end positions were identified based on a ~70% drop in read density downstream of the stop codon relative to the upstream coding region (Supplemental Methods). (**b**) Mapping diffuse 3′ end tapering in *Syn*. Normalized read densities for a random subset of 10 diffuse ends were mapped for 100-nucleotide (nt) sliding windows around the diffuse end position. Position 0 denotes the window ending at the diffuse end position. Differently colored/patterned traces represent the 10 randomly sampled diffuse ends. (**c**) Analysis of undetected intrinsic termination at diffuse ends. TransTermHP-predicted intrinsic terminators in the downstream intergenic region of diffuse ends were identified. Only terminators that passed a high confidence filter were included in the analysis (Supplemental Methods). The intergenic region is defined as the sequence from the stop codon of the gene under consideration to the next start codon on the same strand (limited to 1 kilobase if longer). One kilobase gray box is drawn to scale. (**d**) Frequency of diffuse ends with a high-confidence intrinsic terminator predicted in the downstream intergenic region. (**e**) Nucleotide content in the 201-nt region upstream of defined and diffuse TU ends (“defined,” “diffuse”) and the 3′ ends of all internal operon genes (i.e., within TUs but not at TU 3′ end), used to control for general gene-end effects (all). Box plots show A/T/C/G content distributions; whiskers represent the 5th and 95th percentiles. Gray dotted lines indicate background genomic nucleotide frequencies. Statistical significance was assessed using unpaired two-sided Welch’s *t*-tests (n.s. = not significant; * = *P* < 0.05; ** = *P* < 0.01; *** = *P* < 0.001). Kb = kilobase; min = minimum.

We also uncovered a class of transcript 3′ ends that taper off over many positions indicating RNA isoform heterogeneity. In *Syn*, 46% of TU ends tapered diffusely (*n* = 349), compared to just 25% (*n* = 243) in *Eco* and 7% (*n* = 75) in *Bsu* (Fig. 2a). In *Syn*, these ends typically run 100–400 nt until read density drops to ~30% of the level within the gene body (Fig. 4a and b; Fig. S3; Supplemental Methods). Only 4% of *Syn* genes with diffuse ends had predicted high-confidence intrinsic terminators downstream (*n* = 14/349) (Fig. 4c and d; Supplemental Methods), indicating that diffuse ends likely are not generated by processing from intrinsic terminators and arise through a distinct mechanism.

This tapering profile could result from factor-based termination mechanisms like Rho, which acts over a range of positions (1). Strikingly, diffuse ends were most abundant in *Syn*, the only species tested that lacks known termination factors to explain this profile. Alternatively, diffuse ends could arise from incomplete exoribonucleolytic decay giving a wide distribution of end positions or from unknown termination mechanism(s) that do not rely on a single point of termination. Because nucleotide enrichment signatures are known to be associated with shared termination mechanisms like the pyrimidine-rich signature of *rut* sites around Rho-terminated transcript ends (2, 5), we looked at nucleotide preferences at *Syn* diffuse ends (Supplemental Methods). However, we did not see strong preferences that would help to elucidate their termination mechanism (Fig. S4), apart from a very modest preference for adenine depletion and guanine enrichment in the 201 nt upstream of diffuse ends (Fig. 4e).

### Examination of noncanonical termination factors

Our data demonstrate that most TUs (~80%) in *Syn* are not terminated by the canonical bacterial mechanisms. However, alternative transcription termination factors have been documented in archaea, eukaryotes, and other bacterial contexts. We, therefore, investigated whether similar mechanisms could contribute to cyanobacterial termination.

**Fig 5:**
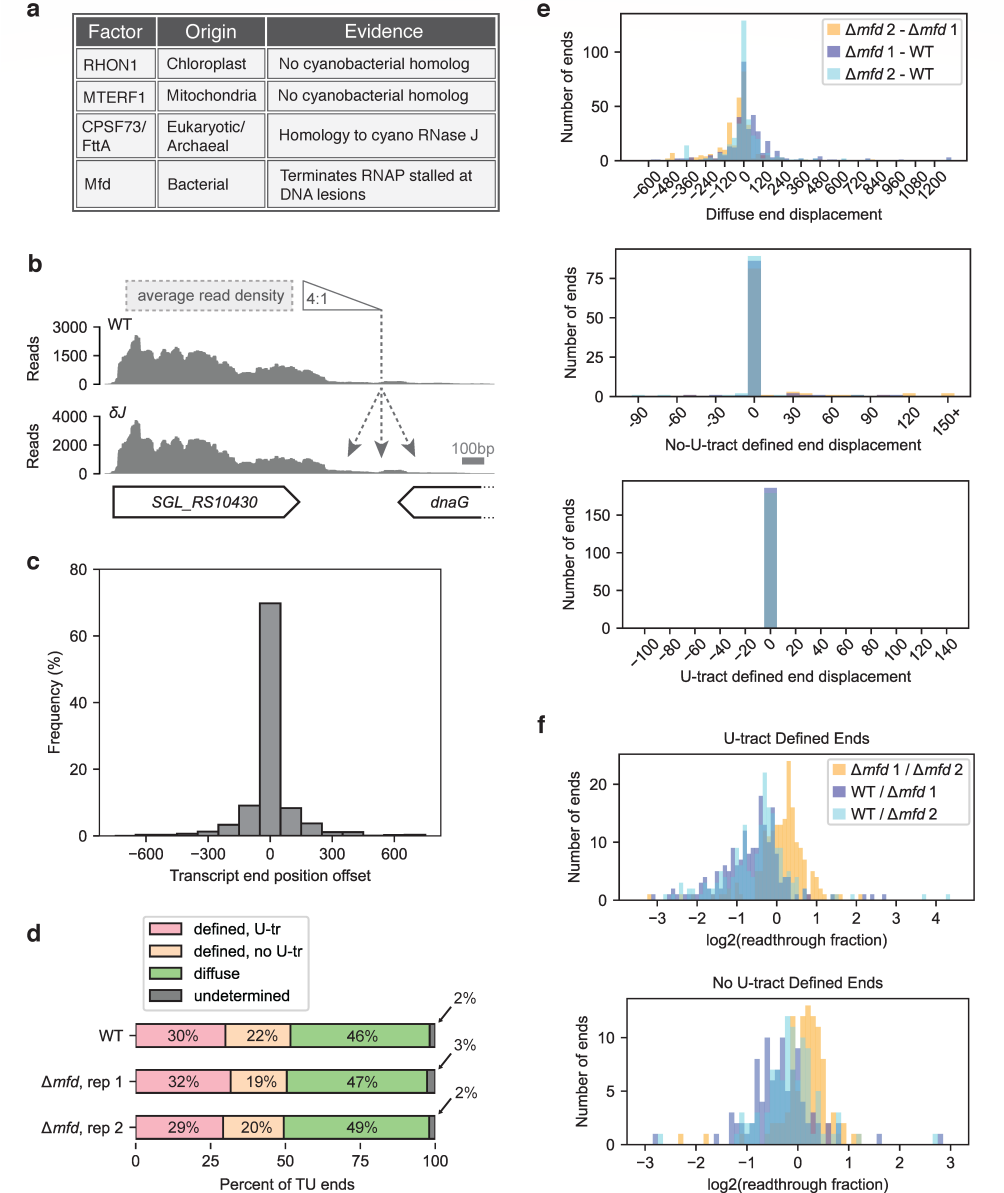
Examination of candidate factor-dependent termination mechanisms in cyanobacteria. (**a**) Summary of candidate termination factors. Results of BLAST-based homology searches comparing known eukaryotic, archaeal, and organellar factors to the *Synechococcus elongatus* PCC 7942 (*Syn*) genome are listed, as well as rationale for testing bacterial Mfd. (**b**) RNA-seq data analysis to test for evidence of RNase J termination factor activity using wild-type (WT) and RNase J-depleted *Synechocystis* sp. PCC 6803. Read density in the coding sequence is compared to downstream read densities to identify the window where a drop exceeding fourfold is achieved, defining the transcript end (Supplemental Methods). The offset in TU end positions between data sets is calculated as Position_depletion_ − Position_WT_. Gray bar shows *x*-axis scale. (**c**) Distribution of TU end position offsets in RNase J-depleted *Synechocystis* versus WT. (**d**) Distribution of TU end types identified by automated Rend-seq classification (Supplemental Methods) for two biological replicates (reps) of Δ*mfd*. End-type distributions were consistent with WT *Syn* for a similar number of genes analyzed (*n* = 1,238 genes, 656 TUs for Δ*mfd* rep 1; *n* = 1,264 genes, 692 TUs for Δ*mfd* rep 2). (**e**) Distribution of TU end position offsets between WT *Syn* and Δ*mfd* reps for U-tract defined ends, non-U-tract defined ends, and diffuse ends. Offsets were calculated as Position_Δ*mfd*_ − Position_WT_ (Supplemental Methods). (**f**) Fold-change in transcriptional readthrough at defined ends with or without U-tracts. Readthrough (RT) fractions were calculated as RT_WT_/RT_Δ*mfd*_ (WT vs Δ*mfd* reps) or RT_Δ*mfd* rep 1_/RT_Δ*mfd* rep 2_ (between reps) (for RT calculation, see Supplemental Methods). Cyano = cyanobacterial; δJ = RNase J-depleted *Synechocystis*; U-tr = U-tract; WT = wild type; Δ*mfd* = *Syn* Mfd knockout; Δ*mfd* 1 = Δ*mfd* rep 1; Δ*mfd* 2 = Δ*mfd* rep 2.

Several termination factors have been reported in eukaryotic organelles of bacterial origin. In chloroplasts, RHON1 is known to mediate some plastid transcription termination in a Rho-like manner (76, 77). However, no RHON1 homologs in *Syn* were found via a protein-protein BLAST. Expanding this search to the entire cyanobacterial phylum returned seven low-confidence hits from six unique genomes with weak similarity to RHON1’s C-terminal region (30–54-amino acid [aa] fragments with 59.5%–60.0% identity [*E* values 2 × 10^−4^–0.026]), suggesting they are unlikely to represent functional homologs (Fig. 5a; Supplemental Methods). We also did not detect cyanobacterial homologs of the mitochondrial transcription termination factor MTERF1 (78) (Fig. 5a; Supplemental Methods).

Another candidate is FttA/CPSF73, the conserved termination factor for archaea and eukaryotic Pol II systems (79, 80). Protein-protein BLAST revealed FttA homology to an ~100 aa fragment of *Synechococcus elongatus* RNase J (Fig. 5a; Supplemental Methods). More extensive homology to RNase J was identified using DELTA-BLAST, a tool optimized to detect remote protein homologs (81) (Supplemental Methods). This 421-aa fragment of *S. elongatus* RNase J shared 16.6% identity to FttA (*E* value 2 × 10^−26^) and spanned the full MβL and β-CASPase domains that form the FttA nucleolytic active site (82). While RNase J was initially identified as a ribonuclease in *Bsu* (83, 84), which encodes paralogs RNase J1 and J2, RNase J1 was recently shown to terminate stalled ECs through a torpedo mechanism that is functionally analogous to FttA/CPSF73-dependent termination (83).

These findings suggest that RNase J could act as a general cyanobacterial termination factor, mediating an archaeal/eukaryotic-like termination pathway in bacteria. To test this possibility, we reanalyzed RNA-seq data from a *Synechocystis* sp. PCC 6803 RNase J depletion strain (84). We hypothesized that the depletion of a general termination factor would cause widespread transcriptional readthrough leading to downstream shifts in transcript end positions. However, ~40% of transcripts showed no offset in end position between wild-type (WT) and RNase J-depleted *Synechocystis*, and the overall offset distribution was centered at zero, suggesting any shifts were largely noise-driven (Fig. 5b and c; Supplemental Methods). These findings do not support a role for RNase J as a global cyanobacterial transcription termination factor.

Finally, we assessed whether the transcription-repair coupling factor Mfd contributes to TU end formation in *Syn* (Fig. 5a). Mfd-mediated transcription termination is the only other well-characterized bacterial termination pathway, but its activity is associated with resolving ECs stalled at DNA lesions genome-wide, not with producing TU ends (1, 14, 85). Were Mfd required for termination at TU ends, its loss should cause downstream offsets in transcript end positions relative to WT. To test this possibility, we generated an Mfd knockout strain (Δ*mfd*) and performed Rend-seq on two biological replicates (reps) (Materials and Methods; Supplemental Methods; Fig. S4 and S5). End-class distributions were consistent with WT for a similar number of genes analyzed (Fig. 5d; Supplemental Methods; Table S1), and Mfd deletion caused no substantial offsets in TU end positions across classes (Fig. 5e). However, there was an overall increase in transcriptional readthrough at defined ends, with the most pronounced effect at those with U-tracts. Among defined ends with U-tracts in both WT and Δ*mfd* data sets for which readthrough fold-changes could be calculated, nearly all showed increased readthrough upon Mfd deletion (Fig. 5f; Supplemental Methods). 25% of these ends (average of replicates) exhibited ≥2-fold increases in readthrough, and half showed at least 1.35-fold increases (Fig. 5f). The effect was less pronounced at defined ends lacking U-tracts (Fig. 5f), consistent with their elevated baseline readthrough in WT (Fig. S7). Thus, Mfd is not required for termination like canonical factors such as Rho, as its loss does not cause complete readthrough or major shifts in end position. Rather, these results could suggest a previously unrecognized role for Mfd in TU end formation. Mfd may act at the observed RNA 3′ ends, potentially stimulating intrinsic termination efficiency, or in the downstream region, where Mfd-dependent termination events are then processed back to the observed endpoints. We did not observe substantial changes in gene expression upon Mfd deletion, so indirect effects are unlikely to contribute to the readthrough changes.

## DISCUSSION

Through precision mapping of TU termini, our work reveals that intrinsic termination plays a minor role in terminating transcription in the freshwater cyanobacterium *Syn*. Defined ends in *Syn* that lack intrinsic terminators are not highly structured, leaving their mechanism of formation and stabilization unclear. This contrasts with the predominance of intrinsic terminators in *Bsu* and the prevalence of RNA secondary structures at defined ends in *Eco*. In *Syn*, diffuse 3′ ends—broad, tapering regions that ramp down long after genes without known terminators—were detected at nearly half of all TUs. There were no strong nucleotide biases across these regions, and the functional relevance, if any, of weak preferences remains unknown. Finally, Mfd loss increased apparent transcriptional readthrough at intrinsic sites and, to a lesser extent, other defined ends without shifting the position of termination, suggesting a role in stimulating transcription termination at or downstream of defined ends. The approximately 80% of TU ends that are not formed by intrinsic termination suggest that additional, unknown termination mechanisms may operate in cyanobacteria.

Mfd is typically associated with an independent termination pathway for removing ECs stalled at sites of DNA damage (1, 14, 85). The connection to DNA damage is notable in the context of cyanobacteria, whose photosynthetic lifestyles involve frequent exposure to UV radiation that produces high levels of photodamage to their genomes, including thymine dimers (86, 87). Greater reliance on Mfd termination in response to elevated DNA lesions could explain the widespread increase in RNA levels we observed downstream of defined 3′ ends upon Mfd deletion (Fig. 5f), as transcript ends generated by Mfd-dependent termination would likely be processed back to stabilizing 3′ ends. Alternatively, Mfd could act directly at defined 3′ ends, which may be hotspots of UV-induced thymine dimer lesions due to the enrichment of coding-strand Ts in the U-tract region, consistent with our observation of increased thymine content at defined ends (Fig. 4e). However, given the low frequency of such lesions (5–40 per megabase of DNA) (86) and the consistency in termination point required to produce a defined end from many uniform-length RNA isoforms, a model whereby Mfd broadly promotes termination and transcript ends are processed back to stabilizing 3′ ends appears more likely. Further characterization of the frequency and position of Mfd-dependent termination will be required to uncover Mfd’s role in shaping cyanobacterial transcript ends.

The possibility remains that U-tract-less defined and diffuse TU ends do not reflect transcription termination sites, but rather other processes that can generate transcript 3′ ends. For example, endoribonucleolytic cleavage and/or exoribonucleolytic trimming from downstream termination sites could obscure the true point of termination. In some cases, strong transcriptional pausing alone could appear as a TU end in our data. Additionally, the RNA structures uncovered at 46% of U-tract-less defined ends could be involved in other types of regulation. RNA hairpins are known to stabilize transcripts against decay, guide ribonuclease processing, mediate RNA-RNA interactions, and drive hairpin-stabilized transcriptional pausing (2, 67). Unstructured defined ends and diffuse ends may also be stabilized by small RNAs or RNA-binding proteins. A combination of such processes—e.g., endonucleolytic cleavage followed by selective stabilization—could underlie formation of these non-canonical ends. This possibility represents an important caveat to interpreting the sequence features identified at these ends in our work. Regardless, canonical intrinsic termination remains an unlikely explanation for these end categories given the marked absence of predicted terminators downstream (Fig. 3d and 4d).

The 5′-TG-3′ dinucleotide motif common to some defined 3′ ends (Fig. 3a) partially overlaps with the consensus bacterial elemental pause sequence—a hairpin-independent pause signal—particularly the Y_−1_G_+1_ motif consisting of a pyrimidine at the nascent RNA 3′ end and guanine at the incoming nucleotide position (67). Another component of the consensus sequence with a pronounced effect on pausing, G_−10_, is not present in our data (67). We have several hypotheses for the origin of this motif. First, it could arise from nascent RNA associated with paused RNAPs. Alternatively, these 3′ ends might be generated by pause-induced RNA release that is distinct from previously described termination mechanisms. It could also represent a ribonucleolytic processing signal that may or may not be related to termination.

Several mechanisms could explain termination of TUs with diffuse 3′ ends. First, RNAP may exhibit reduced processivity in the tapering region, pausing frequently and increasing termination likelihood. Additionally, an uncharacterized termination factor could be responsible. One candidate is RNase J, which is homologous to the archaeal termination factor FttA (Fig. 5a) that recognizes U-rich RNA (82, 88). Cyanobacteria represent a deep bacterial lineage that branches directly from a common ancestor of Archaea-Eukarya and Bacteria (89), raising the possibility that a termination factor in this phylum may more closely resemble that of archaea and eukaryotes than the canonical Rho pathway found in many bacteria. We did not observe clear evidence that RNase J acts as a global termination factor in *Synechocystis*; however, the depletion strain was not fully segregated, with PCR confirming persistence of WT *rnj* alleles (84), suggesting incomplete functional depletion. Clearer RNase J depletion in future studies may uncover stronger impacts on transcription termination.

It is also possible that cyanobacterial RNAP recognizes a non-canonical intrinsic signal, for example, potentially only a U-tract without an accompanying hairpin. It was previously shown that a stretch of uridines is sufficient to pause and induce clamp opening of *Eco* RNAP, which causes nascent RNA release (90). This reaction was importantly independent of any upstream hairpin structure. Such a signal could account for RNA release at the less structured defined ends with U-tracts (Fig. 2c). Another structure-independent pause signal capable of inducing clamp opening and triggering RNA release could also explain defined ends lacking U-tracts, a broadly unstructured end class in our data (Fig. 3b), and possibly also diffuse ends.

The large fraction of *Syn* TUs with diffuse end positions may indicate that only certain genes benefit from having well-defined transcript 3′ ends. We speculated that genes encoding photosystem components (91), which are tightly regulated to adapt the photosynthetic apparatus to changing environmental conditions (e.g., light intensity/quality) (92, 93), may be more likely to have defined transcript boundaries. Indeed, the frequency of defined ends was significantly higher among photosystem transcripts (76%; *P* = 0.05, Fischer’s exact test) compared to the background frequency across the genome (Supplemental Methods). Consistency in the 3′ end of these transcripts could facilitate precise regulation of exonucleolytic decay and thus RNA levels. However, in a broader search for gene functions associated with defined or diffuse TU ends using functional categorizations based on gene orthology (94), we did not identify any significantly enriched or depleted functions for either end type (Supplemental Methods).

Practically, our work provides a useful resource for bioengineering (58). The predictability and efficiency of cyanobacterial metabolic engineering have remained limited, partly due to poorer characterization of genetic parts (48, 66). Our work addresses this gap by vastly expanding the repertoire of functionally characterized intrinsic terminators. We identified 225 defined ends with U-tracts in *Syn* spanning a wide range of predicted efficiencies, including 167 putative terminators that have evidence of both an upstream hairpin and U-tract (Fig. 2; see Table S2 for list of these ends and their efficiencies). Although *in vivo* termination efficiency estimates can be influenced by post-transcriptional processes like exonucleolytic trimming (7), this data set provides a valuable starting point for building tunable, precisely controlled cyanobacterial expression systems.

In summary, the absence of canonical intrinsic terminators in *Syn* reveals a broad, largely uncharted landscape of regulatory biology, potentially shaped by its unique photosynthetic lifestyle, high genomic ploidy, and circadian rhythms. As a lineage that has persisted since life’s earliest days, cyanobacteria represent a largely untapped frontier that is ripe for discoveries that may reshape our understanding of transcription termination and provide new, yet ancient, answers to fundamental questions about the expression codes of life.

## MATERIALS AND METHODS

### Growth conditions

*Syn* WT and Δ*mfd* cells were cultured in BG-11 media optimized for cyanobacteria (Gibco, Catalog No. A1379901), and Δ*mfd* cultures were supplemented with 5 µg/mL kanamycin. Cells were grown at 30°C with 120 rpm of shaking under a 12 h:12 h (12:12) light:dark diel cycle with 140–160 µE light intensity (GE 40 Watt, 24” non-dimmable balanced light spectrum LED grow light).

For Rend-seq, 25 mL cultures were inoculated from WT (JAC9) and Δ*mfd* (JAC27) glycerol stocks in 125 mL flasks. When cultures reached ~mid-exponential phase (OD_730_ = 0.26 [WT] to 0.35 [Δ*mfd*]) 15 days later, they were back-diluted in a total volume of 250 mL in 2.8 L flasks such that 4 doublings were required to reach an OD_730_ = 0.05, the beginning of exponential phase in our growth conditions (target OD_730_ in back-dilution = 0.003125). One flask of WT and two biological replicate flasks of Δ*mfd* (Δ*mfd* reps 1 and 2) were prepared. When cultures had grown up again to exponential phase (4 days later), cells were collected at hour 7 in the 12:12 light:dark diel cycle by pelleting 3 × 35–40 mL of cells at OD_730_ = 0.14–0.22 for 10 min at 4,000 rpm at 4°C (OD_730_ WT = 0.22; Δ*mfd* rep 1 = 0.14; Δ*mfd* rep 2 = 0.19). After pelleting, supernatants were removed, and the cell pellets were flash frozen in liquid nitrogen and stored at −80°C until ready for use in RNA extraction (no longer than 1 month).

### Construction of Mfd knockout strain

To knock out endogenous Mfd (gene name: *Synpcc7942_1326*), a knockout plasmid was synthesized using TWIST Bioscience’s clonal gene service. A knockout fragment targeting *Synpcc7942_1326* for replacement with a kanamycin resistance (KanR) cassette was inserted into the pTwist Amp High Copy backbone (high copy cloning vector with pMB1 origin of replication and an ampicillin resistance cassette). This knockout fragment contained the promoter and coding sequence of the KanR gene *aphI* encoding aminoglycoside 3′-phosphotransferase derived from the cyanobacterial cloning vector pCV0049 designed by Taton et al. (95) flanked by homology arms targeting the *Synpcc7942_1326* locus. The left and right homology arms comprised the 500 bp sequences immediately flanking the CDS of *Synpcc7942_1326*. The resulting plasmid, pJAC041, was resuspended in 10 mM Tris, pH 8, to a final concentration of 100 ng/µL.

Transformation of pJAC041 into WT *Syn* cells was performed using an established protocol (96) with slight modifications for lower speed/longer length spins to avoid breaking off transformation pili during cell harvesting as suggested (97). Briefly, a 25 mL liquid WT *Syn* culture was grown to an OD_730_ of 0.35 under the culturing conditions described in the above section, except constant light was employed, and the culture was used directly after outgrowth from the glycerol stock. A volume of 5 mL of cells was pelleted by centrifugation at 2,000 rpm for 20 min at room temperature. Cells were resuspended in 5 mL of 10 mM NaCl, pelleted by centrifugation as before, and then resuspended in 0.3 mL BG-11 and transferred to microfuge tubes. One hundred nanograms (1 µL) of plasmid DNA or 1 µL H_2_O (no DNA control) was added, and the tubes were briefly vortexed and inverted several times to mix. Tubes were then wrapped in aluminum foil to block light exposure and incubated in the dark overnight (21 h) under the culturing conditions described. The next day, the entire cell suspension was plated on a pre-warmed selective BG-11 agar plate (1.5% agar, 5 µg/mL kanamycin) and incubated at 30°C under constant light (~120–200 µE). Once colonies appeared (6 days later), a patch of transformant cells was restreaked onto a fresh selective plate to ensure full chromosomal segregation. When colonies appeared on that plate (7 days later), a single colony from the second plate was again streaked out onto a new plate. When single colonies appeared on the third plate (7 days later), 5 mL BG-11 liquid cultures supplemented with 5 µg/mL kanamycin were inoculated from single colonies in test tubes. Cultures were grown under the conditions described in the above section, except with constant light. Once cultures were visibly green (6 days later), chromosomal PCR was performed to confirm successful integration of the KanR gene at the *mfd* locus using a previously described protocol (98). Briefly, 1 mL of culture was pelleted in a microfuge tube at 14,000 *g* for 4 min at room temperature. The supernatant was removed, and pellets were resuspended in 100 µL TE, transferred to fresh tubes, heated at 100°C for 3 min on a heat block, and centrifuged as before to remove cellular debris. The supernatant was transferred to a new tube, and then 2 µL of cleared supernatant was used in 25 µL PCRs with Phusion High-Fidelity DNA polymerase (NEB, Catalog No. M0530L). PCR was performed to detect the left integration junction (primers oJAC259 + oJAC260; 748 bp), right integration junction (oJAC261 + oJAC262; 778 bp), and across the insertion site (oJAC259 + oJAC262; 2,159 bp for the KanR insertion or 4,707 bp for the WT *Synpcc7942_1326* allele). The cross-site PCR used a 2 min 22 s extension time to allow amplification of the larger WT product, if present. PCR confirmed successful integration and full chromosomal segregation of the knockout construct (Fig. S5). The Mfd knockout strain (GLB637) did not exhibit a noticeable growth defect under the culturing conditions used.

### Rend-seq and computational analysis

Rend-seq was performed as previously described (8, 99). For further detail on the protocol and computational analysis, refer to the Supplemental Methods.

## Data Availability

All sequencing data (raw FASTQ and shadow-removed WIG files) have been deposited in the Gene Expression Omnibus under accession number GSE309256 (BioProject PRJNA1334990).

## SUPPLEMENTAL MATERIAL

Supplemental Figures msystems.01581-25-s0001.pdf Figures S1-S7.

Supplemental Methods msystems.01581-25-s0002.docx Detailed methods.

Table S1 msystems.01581-25-s0003.xlsx End classifications for all genes and data sets.

Table S2 msystems.01581-25-s0004.xlsx Features associated with defined ends.

ASM does not own the copyrights to Supplemental Material that may be linked to, or accessed through, an article. The authors have granted ASM a non-exclusive, world-wide license to publish the Supplemental Material files. Please contact the corresponding author directly for reuse.
