# Supplementary material for: Canonical transcription termination mechanisms explain a minority of operons in cyanobacteria
Source: mSystems. 2026 May 18;11(6):e01581-25. doi: 10.1128/msystems.01581-25 (PMC13289151; doi:10.1128/msystems.01581-25)
Supplement: Supplemental Figures — Figures S1-S7. [file msystems.01581-25-s0001.pdf]

Supplementary Figures

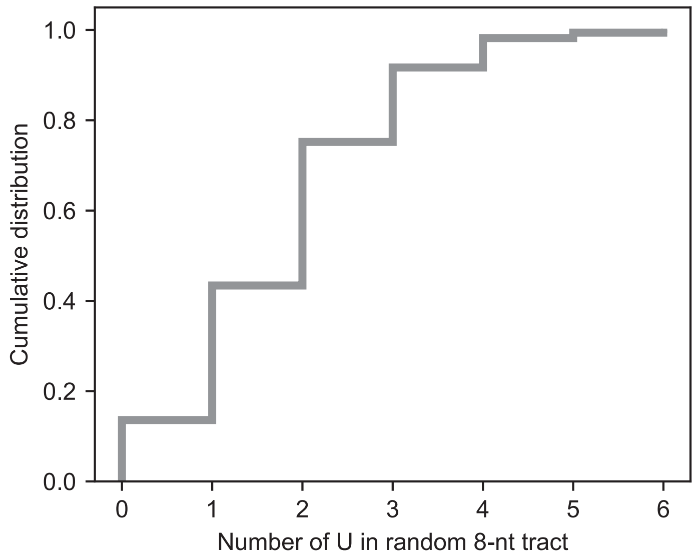

Supplementary Figure 1. Cumulative distribution of uridine counts in 1,000 random 8-nucleotide (nt) sequence tracts in *Synechococcus elongatus* PCC 7942. Sequences were sampled evenly from the forward and reverse strands. A threshold of ≥ 4 uridines (8% chance of occurring at random) was chosen to define U-tracts. There is a 2% chance of ≥ 5 uridines occurring at random in an 8-nt tract and a 25% chance of ≥ 3 uridines.

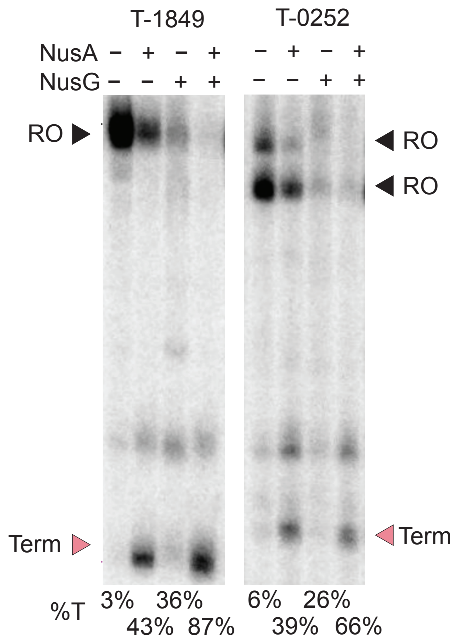

Supplementary Figure 2. Multi-round *in vitro* transcription termination assays for the intrinsic terminators downstream of the genes *Synpcc7942_1849* (left) and *Synpcc7942_0252* (right). Experiments were performed in the presence (+) or absence (-) of NusA and/or NusG as indicated (**Supplementary Methods**). Positions of terminated (Term, pink arrow) and run-off (RO, black arrow) product are marked. Termination efficiencies (%T) for each terminator under the specified conditions are indicated below the lanes (**Supplementary Methods**).

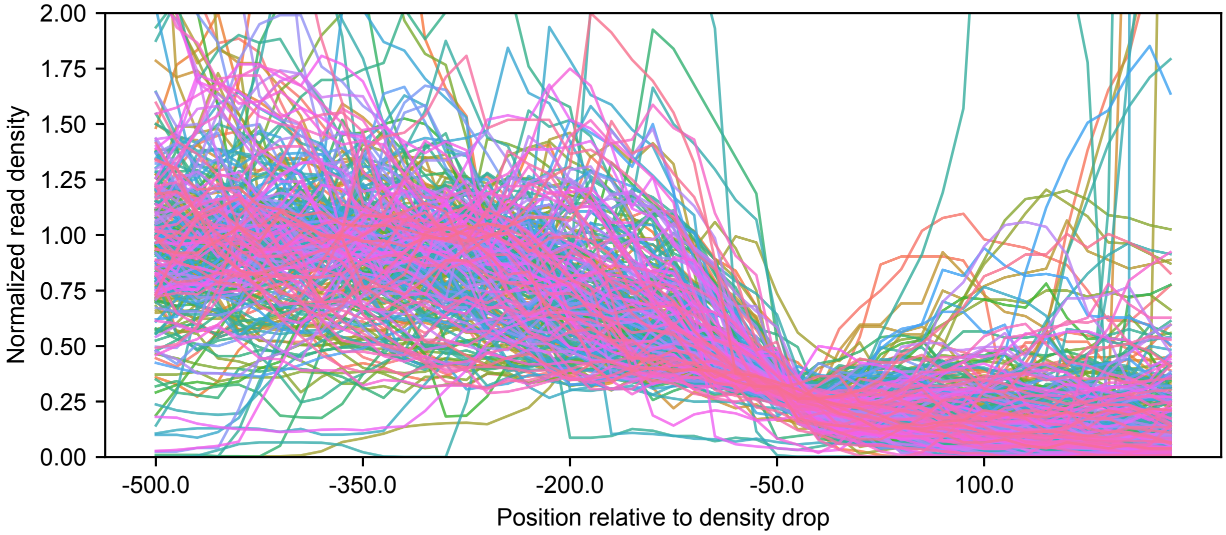

Supplementary Figure 3. Global tapering behavior at *Synechococcus elongatus* PCC 7942 diffuse TU ends. Normalized read densities were calculated in a fixed window around diffuse end positions as described in the **Supplementary Methods**. All diffuse ends are shown except those that contain multiple 3′ peaks (diffuse peaks) (n=274).

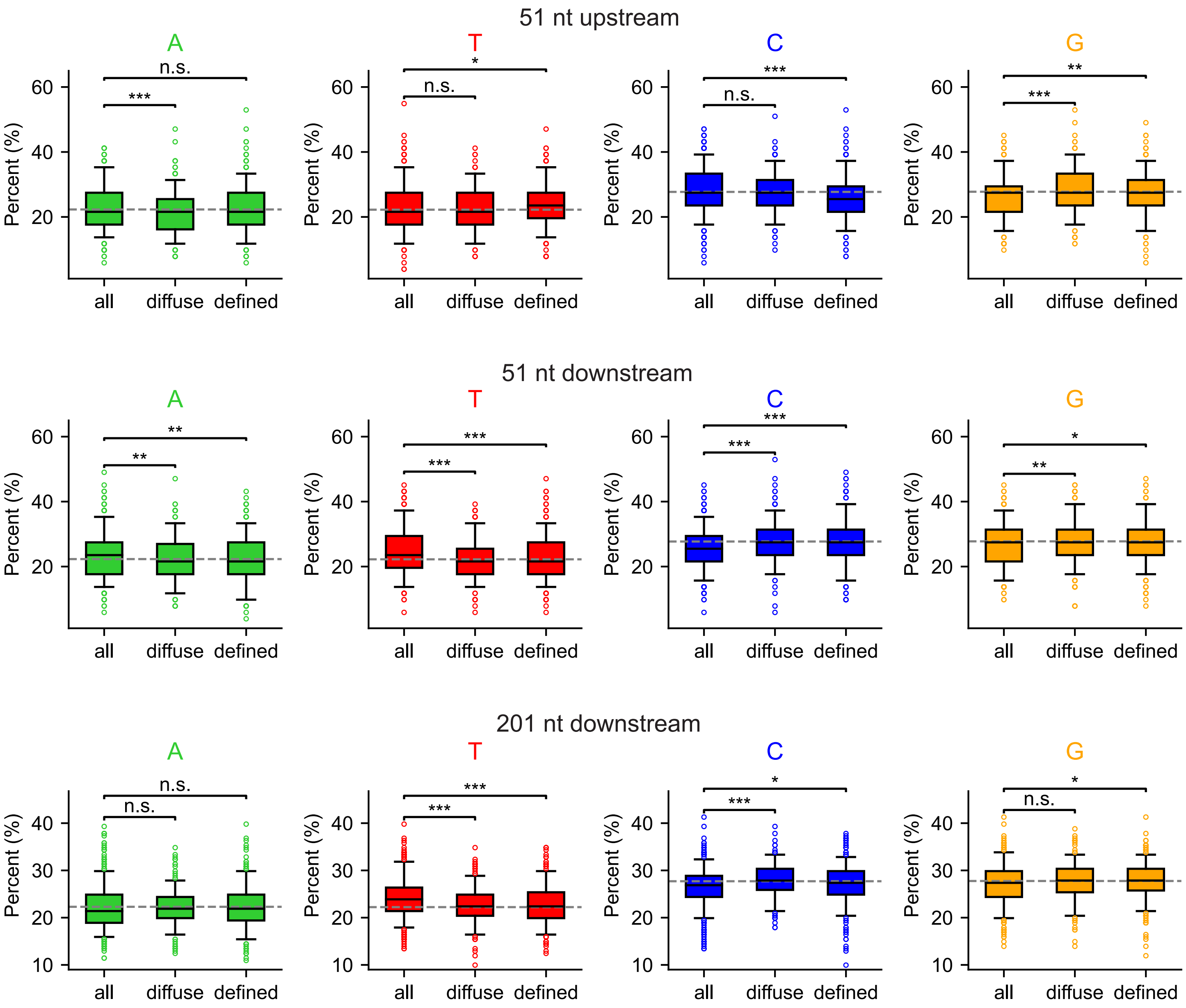

Supplementary Figure 4. Nucleotide (nt) composition analysis in upstream 51-nt window and downstream 51-nt and 201-nt windows flanking diffuse TU ends in *Synechococcus elongatus* PCC 7942. Nucleotide content in the specified windows was computed for defined and diffuse TU ends ("defined", "diffuse") and the 3′ ends of all internal operon genes (i.e., within TUs but not at TU 3′ end), used to control for general gene-end effects ("all"). Box plots show A/T/C/G content distributions; whiskers represent the 5th and 95th percentiles. Gray dotted lines indicate background genomic nucleotide frequencies. Statistical significance was assessed using unpaired two-sided Welch's t-tests (n.s. = not significant; * = $p < 0.05$; ** = $p < 0.01$; *** = $p < 0.001$). Biases observed in the 51-nt upstream window mirror those in the 201-nt upstream window but are less pronounced. No clear biases were detected downstream of diffuse TU ends.

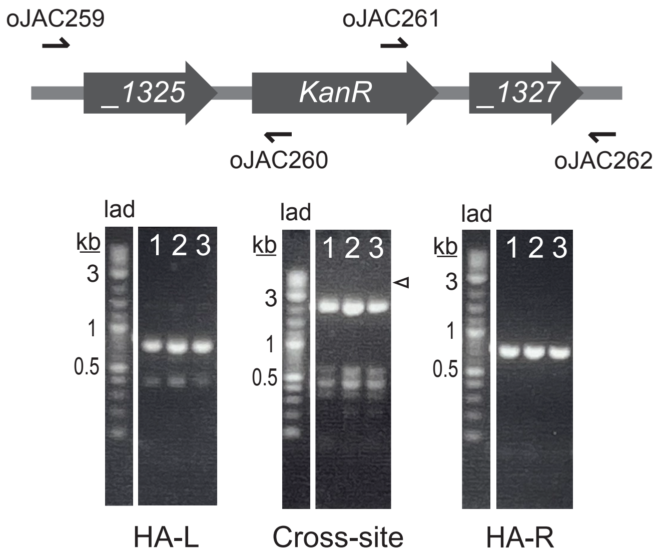

Supplementary Figure 5. Chromosomal PCR confirms Δ*mfd* construction. PCR product sizes are as expected (see **Materials and Methods**) for the left junction, right junction, and cross-site reaction on the three tested colonies, confirming successful knockout strain construction. Black unfilled triangle to the right of the cross-site reaction gel indicates the position where the WT *mfd* allele, if present, would produce a PCR product. Colony 3 was selected as the strain representative for use in Rend-seq. HA-L = left homology arm junction; HA-R = right homology arm junction; *KanR* = kanamycin resistance gene (*aphI*); *_1325* = *Synpcc7942_1325*; *_1327* = *Synpcc7942_1327*; lad = Quick-Load® 1 kb Plus DNA Ladder (NEB, Catalog No. N0469S); WT = wild-type.

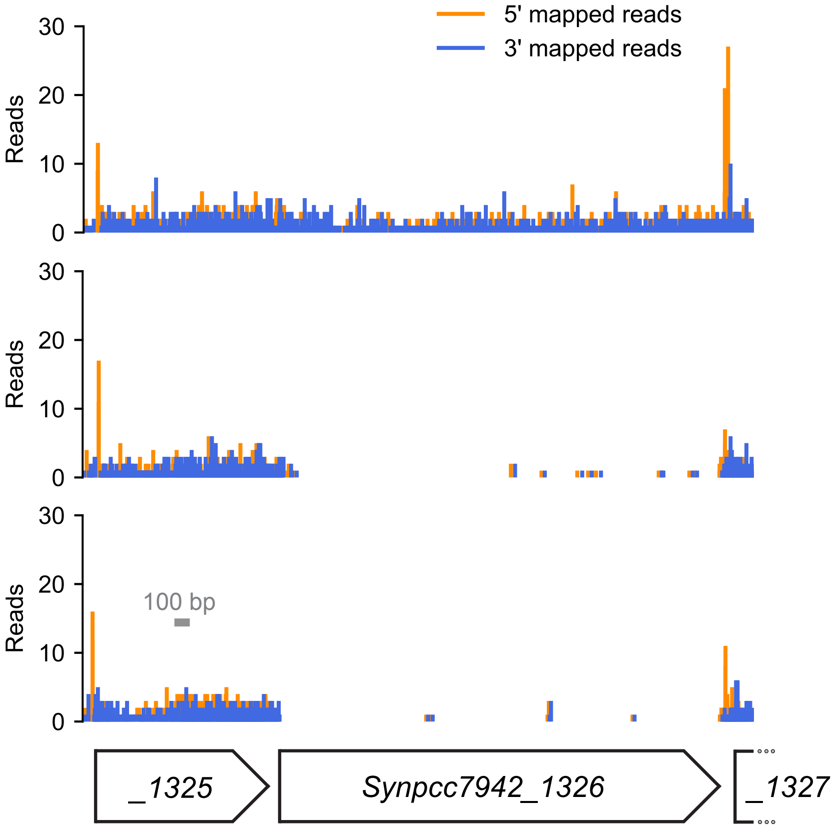

Supplementary Figure 6. Mfd knockout confirmed at the RNA level in *Synechococcus elongatus* PCC 7942 (*Syn*). Rend-seq traces for *Synpcc7942_1326* (*mfd*) and the surrounding genes demonstrate successful transcript-level knockout in Δ*mfd* replicates (reps). Top trace corresponds to wild-type *Syn*, middle trace is Δ*mfd* rep 1, and bottom trace is Δ*mfd* rep 2. Gray bar shows x-axis scale. *_1325* = *Synpcc7942_1325*; *_1327* = *Synpcc7942_1327*.

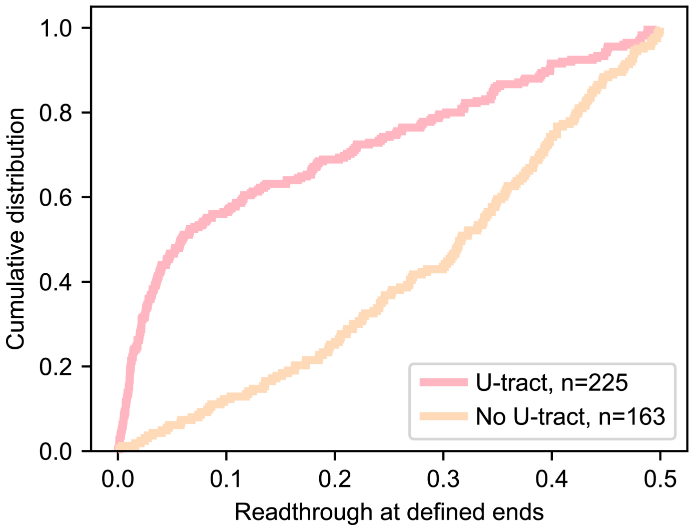

Supplementary Figure 7. Transcriptional readthrough at defined ends with and without U-tracts. Cumulative distributions of readthrough values (calculated as described in **Supplementary Methods**) show elevated baseline readthrough at defined ends lacking U-tracts compared to those with U-tracts.
