# Supplementary material for: Canonical transcription termination mechanisms explain a minority of operons in cyanobacteria
Source: mSystems. 2026 May 18;11(6):e01581-25. doi: 10.1128/msystems.01581-25 (PMC13289151; doi:10.1128/msystems.01581-25)
Supplement: Supplemental Methods — Detailed methods. [file msystems.01581-25-s0002.docx]

**Supplementary Methods**

*RNA Extraction.*

To extract RNA, cell pellets were thawed on ice in 1 mL phenol stop media (6.5:1 BG-11:phenol stop solution; phenol stop solution is composed of 30:18:1:1 100% ethanol:H_2_O:phenol:0.5 M EDTA). One pellet from each Δ*mfd* biological replicate and two WT pellets (technical replicates) were processed, for a total of 4 samples. Thawed pellets were vortexed briefly and centrifuged at 4,000 rpm for 5 min at 4ºC, then resuspended in 0.1 mL of TE buffer per 10 mL of original culture at OD_730_ ~0.2 (350–400 µL TE). NEBExpress® T4 lysozyme (NEB, Catalog No. P8115L) was added at 10 µL per 0.1 mL of resuspended cells (35-45 µL), and samples were incubated at room temperature for 5 min with gentle shaking (300 rpm). Following enzymatic lysis, 2 mL of Buffer RLT (Qiagen) with beta-mercaptoethanol (β-ME) (10 µL β-ME per 1 mL Buffer RLT) were added and samples were vortexed, followed by addition of 2 mL of 70% ethanol. Samples were vortexed, and the resulting ~4.5 mL lysates were loaded onto RNeasy Midi columns (Qiagen, Catalog No. 75144). RNA was column purified per the manufacturer’s instructions, eluted in 150 µL of nuclease-free H_2_O, and then further purified using a 2x SPRI bead cleanup. Briefly, 2 volumes of SPRI beads were added to each RNA sample, mixed thoroughly by pipetting, and incubated for 5 min at room temperature to allow binding of RNA to the magnetic beads. Samples were then placed on a magnetic rack until the solution cleared (~5 min), and the supernatant was carefully removed. The beads were washed twice with 1 mL of 80% ethanol on the magnetic rack, incubating for 30 sec each time. Pellets were air-dried on the magnetic rack (~10 min, until beads appeared slightly cracked), and then RNA was eluted by resuspending beads in a small volume (20-50 µL) of nuclease-free H_2_O, incubating for 5 min at room temperature, and placing on the magnetic rack for 5 min. The supernatant was recovered to retrieve purified RNA. To protect RNA, 1 µL of SuperaseIn RNase Inhibitor (Thermo, Catalog No. AM2696) was added, and samples were then treated with Turbo DNase (Invitrogen, Catalog No. AM2239) per the manufacturer’s protocol to remove contaminating genomic DNA. DNase was heat-inactivated per the manufacturer guidelines, and the reactions were purified by another 2x SPRI bead cleanup as described. RNA quality was confirmed via agarose gel electrophoresis. This protocol effectively lysed cyanobacterial cell walls and yielded high amounts of high-quality RNA (20-30 µg per sample after final cleanup). RNA was stored at -80ºC until Rend-seq library preparation.

*Rend-seq Library Preparation*.

To prepare Rend-seq libraries, rRNA was depleted from 19-20 µg total RNA with MICROBexpress (Invitrogen, Catalog No. AM1905) following the manufacturer’s instructions. Two reactions per sample were performed and then pooled so that the treated RNA amount did not exceed the maximum recommended 10 µg/reaction. Reactions were precipitated in isopropanol. RNA was then fragmented using RNA Fragmentation reagents (Thermo, Catalog No. AM8740) by first incubating RNA in 40 µL of 10 mM Tris, pH 7, at 95ºC for 2 min, then adding 4.4 µL 10x Fragmentation Buffer and incubating at 95ºC for 25 sec (incubations were performed in a PCR thermocycler). The reaction was quenched with 5 µL Stop Buffer. Samples were precipitated in isopropanol and resuspended in 5 µL 10 mM Tris, pH 7. To size-select RNA, samples were mixed with an equal volume of 2x TBE-urea loading buffer and run on a 15% TBE-urea gel (Thermo, Catalog No. EC6885BOX) for 65 min at 200 V. RNA fragments of 15-45 nt were excised and purified, then precipitated in isopropanol. RNA was dephosphorylated with T4 polynucleotide kinase (NEB, Catalog No. M0201S) in a 20 µL reaction by incubating at 37ºC for 1 hr, then the reaction was inactivated at 75ºC for 10 min. RNA was precipitated in isopropanol. Linker-1 was then ligated to the 3′ end of 3 picomoles (pmol) of RNA using T4 RNA Ligase 2, truncated K227Q (NEB, Catalog No. M0351L) in a 20 µL reaction containing 25% PEG 8000, T4 RNA Ligase 2 Buffer at 1x, and 100 pmol of Linker-1 by incubating for 2.5 hr at 25ºC. Ligated RNA was precipitated in isopropanol and resuspended in 6 µL 10 mM Tris, pH 7, then mixed with an equal volume of 2x TBE-urea loading buffer. The ligated product was then purified by size excision on a 10% TBE-urea gel (Thermo, Catalog No. EC6875BOX) run for 50 min at 200 V and precipitated in isopropanol. cDNA was generated by reverse transcription (RT) with SuperScript III (Invitrogen, Catalog No. 18080093) by first incubating ligated RNA with 25 pmol of RT primer oCJ485 in 12.5 µL reactions at 65ºC for 5 min. On ice, the remaining reagents (First Strand Buffer at 1x, 20 U SuperaseIn, 5 mM DTT, 0.5 mM dNTPs, and 1 µL SuperScript III) were then added for a final reaction volume of 20 µL. The reactions were incubated at 50ºC for 45 min and then quenched by adding NaOH to a final concentration of 0.1 M and incubating at 95ºC for 15 min to hydrolyze template RNA. An equal volume of 2x TBE-urea loading buffer was added, and cDNA was purified by size excision on a 10% TBE-urea gel run for 80 min at 200 V and precipitated in isopropanol. Purified cDNA was resuspended in 15 µL 10 mM Tris, pH 8. Single-stranded cDNA was then circularized with CircLigase (VWR, Catalog No. 76081-606) and additional reagents (2.5 mM MgCl_2_, 50 µM ATP, CircLigase Buffer at 1x) in a final 20 µL reaction volume by incubating at 60ºC for 1 hr. Next, 1 µL of CircLigase was spiked in, and the reaction was incubated at 60ºC for 1 hr and then inactivated at 80ºC for 10 min. The final libraries were amplified by PCR of circularized cDNA and purified by size excision on an 8% TBE gel (Thermo, Catalog No. EC6215BOX) run for 45 min at 180 V. Amplified libraries were precipitated in isopropanol, resuspended in 11 µL 10 mM Tris, pH 8, and stored at -30ºC until sequencing.

*Sequencing and FASTQ to WIG File Conversion.*

Rend-seq libraries were sequenced on an Illumina NextSeq500 platform using 75-nt single-end reads. The FASTQ files returned were converted to WIG files as follows. 3′ linker sequences were stripped, and Bowtie version 1.0.0 (-m 1) was used for sequence alignment of uniquely mapping reads to the reference *Syn* genome CP000100.1. The 5′ and 3′ (end-specific) mapped reads were added separately and strand-specifically at genomic positions. WIG files for the WT technical replicates were combined and processed together in the remaining data processing steps. Shadow-removed WIG files for *Bsu* and *Eco* that were previously generated^1^ were processed alongside the new *Syn* WIG files in the post-shadow removal steps of the data processing pipeline (see “Data Processing to Generate Shadow-Removed WIG and Peak Sets” below).

*Peak Calling*.

Because Rend-seq enriches for the original 5′ and 3′ ends of RNA, TU ends can be mapped at single-nt resolution by identifying sharp peaks in the data. We called Rend-seq peaks slightly differently than previously reported^1^. For each genomic position, two modified z-scores were computed by comparing the read count at that position to a surrounding 50-nt upstream and downstream window, excluding a 5-nt gap on either side to avoid artifactual effects from contiguous peaks (i.e., peak widths > 1 nt). We refer to these as the upstream z-score and downstream z-score. Prior to z-score calculation, read values within the window were winsorized to mitigate the effect of extreme values by replacing outlier values (> 1.5 standard deviations away from the mean) with the mean of non-outliers. The mean (*avg*) and standard deviation (*stdev*) for the winsorized window was calculated, and the modified z-score (*z*) was computed as:

$z=\frac{reads-avg}{stdev}$ *,*

where *reads* refers to the read counts at the genomic position being assessed. Z-scores were only reported for positions with sufficient position-specific read counts and read density in the surrounding window (read threshold ≥ 10 reads, density threshold ≥ 0.25 reads/nt) to avoid false-positives due to counting noise. For these positions, the final z-score was defined as the minimum of the upstream and downstream z-scores. If one of the two could not be computed (e.g., due to insufficient read density in one of the windows), the available z-score was retained. Because the z-score tracks deviation from the local mean signal, true peaks at TU boundaries should be distinguished relative to both flanking regions and have large z-scores in both windows. Taking the minimum ensures that only positions showing a sharp transition in both directions are retained, improving discrimination of true peaks.

Peaks were then called by thresholding on the z-scores. The inverse cumulative distribution (1-CDF) of z-scores was computed, and a threshold was set where the distribution shows a transition between two populations (peaks vs. non-peaks). A z-score threshold of ≥ 12 was used to call peaks on both the original and shadow-removed WIGs for all *Syn* datasets and ≥ 13 on shadow-removed WIGs for *Bsu* and *Eco* (see “Data Processing to Generate Shadow-Removed WIG and Peak Sets” below).

*Data Processing to Generate Shadow-Removed WIG and Peak Sets.*

The data processing pipeline was as follows. First, peaks were called (see “Peak Calling” above) on WIGs in the 5′ and 3′ channel for the three *Syn* datasets (WT, Δ*mfd* rep 1, Δ*mfd* rep 2). Then, the peaks were collapsed. In Rend-seq data, multiple closely spaced peaks may initially be called in the same channel, such that the peak width for a given genomic region—e.g., at a TU boundary—is > 1 nt. At TU ends, clusters of peaks may represent true 3′ ends produced by limited multi-position intrinsic termination or partial 3′-5′ exoribonucleolytic processing that shifts the transcript end by a few nt. To define TU end positions, peaks within ±5 nt were collapsed to the one with the highest z-score. The next step, shadow removal, also requires collapsed peaks as input. Shadow removal was performed as previously reported^1^. Briefly, this process involves removing regions of elevated read density adjacent to peaks (peak “shadows”) that arise as an artifact of Rend-seq end-enrichment. Peaks were then re-called on the shadow-removed WIGs and collapsed again. These final shadow-removed WIG and peak sets were used for TU end calling (see “End Calling Pipeline” below). Note that *Bsu* and *Eco* WIGs were already shadow-removed^1^ and were thus processed starting from the post-shadow removal steps. The following approximate number of reads (in millions, M) were obtained for the *Syn* datasets after processing: 13.5 M (*Syn* WT), 10.2 M (*Syn* Δ*mfd* rep 1), and 10.7 M (*Syn* Δ*mfd* rep 1). Shadow-removed WIG files confirmed the absence of *mfd* transcript in the Δ*mfd* strain, consistent with a successful knockout (**Supplementary Fig. 6**).

*End Calling Pipeline.*

To automatically identify and classify TU ends in Rend-seq data, we searched the region downstream of each annotated gene (from the annotation file for the complete *Syn* chromosome CP000100.1; *Bsu* chromosome NC_000964.3; and *Eco* chromosome NC_000913.2) for the drop in read density indicative of a transcript endpoint(s). The presence or absence of a single downstream 3′ peak was then used to distinguish between a defined or diffuse transcript end.

To ensure that end classification could be performed confidently, the following criteria were applied. Genes had to be longer than 155 bp, and the winsorized read density (reads above 98^th^ percentile winsorized) across the gene body (excluding 45 bp on either end) had to exceed 0.5 reads/bp. Since 3′ peak detection is limited at low read depth, for genes with a diffuse end classification, we also required that the read depth at the end of the gene (30-105 bp upstream of the stop codon) independently pass a threshold of 0.5 reads/bp. 1,295 genes in WT *Syn*, 2,176 in *Eco*, 2,674 in *Bsu*, 1,238 in Δ*mfd* rep 1, and 1,264 in Δ*mfd* rep 2 were classified. End classification results for all datasets are provided in **Supplementary Table 1**.

Before performing end classification, the set of 3′ peaks identified in each Rend-seq dataset was filtered to generate a set of candidate defined transcription end positions by requiring that read density drop downstream of the peak. For each 3′ peak, the readthrough fraction was determined as previously described^1^. In brief, the readthrough fraction captures the ratio of the read density downstream of the peak to the read density upstream. If a transcript ends at a 3′ peak, the readthrough is expected to be less than 1 due to the drop in read density downstream of the peak. A 3′ peak was therefore included in the set of candidate defined transcription ends if the readthrough fraction was below 0.5.

End classification was then performed as follows. The pipeline alternated between a search for (1) a candidate defined transcription end, and (2) a drop in read density in the region downstream of the stop codon. The search began with a 75-nt window extending 100-175 bp downstream of the stop codon. If the initial downstream window extended into the next TU (marked by a 5′ peak) or past the end of the next gene (marked by a stop codon on the same strand), the regions used for classification were modified as follows. The search for candidate transcription ends was limited to the region between the end of the current gene (10 bp upstream of the stop codon) and the TU boundary (5′ peak or next stop codon position). If no defined end was found, the window for calculating the downstream read density was repositioned to extend 15-90 bp upstream from the TU boundary and trimmed to exclude any overlap with the gene being classified. If the trimmed window was less than 20 bp, the gene was excluded from end classification.

After establishing a downstream window, the region extending from the end of the gene (10 bp upstream of the stop codon) to the end of the downstream window was searched for candidate defined transcription ends. If a candidate defined end was found, a secondary search was performed for any additional 3′ peaks (with any degree of readthrough) between the gene stop codon and candidate defined end. As such additional peaks may represent alternate transcript ends, the end type was recoded to be a “diffuse peak” if additional 3′ peaks were found. In **Figs. 1, 2, 4**, and **5**, the diffuse peaks were counted as diffuse ends. If no additional 3′ peaks were found, an end type of “defined” was assigned to the gene. In some cases, transcription of a gene ended with a 3′ peak, but readthrough could not be defined due to a downstream 5′ peak in close proximity. These genes were classified as having an “undetermined” end type.

If no defined end was found, a diffuse end type was assigned if the read density dropped by at least 3.5-fold between the end of the gene (30-105 bp upstream of the stop codon) and the downstream window. The downstream edge of the window was then recorded as the diffuse end position. If no evidence of a defined or diffuse end was found, the downstream window was stepped downstream by 20 bp, and the two-step search for a defined or diffuse end was repeated with the new window.

This process continued until an end type was assigned, or until the downstream window reached the beginning of the next TU (marked by a 5′ peak) or extended past the stop codon of the following gene on the same strand. At this point, if no evidence of a transcription end was found, the gene was classified as part of a TU (i.e., “TU-internal”).

If at any point the downstream density exceeded the density in the gene by at least 3.5-fold, the end type was assigned to be “undetermined,” as read density is expected to drop or be maintained in the absence of a transcription start site.

*U-tract Analyses.*

To classify defined ends as having a U-tract, we calculated the percent uridine content in the 8 nt immediately preceding and including the 3′ peak position. U-tracts were scored for peaks with at least 4 uridines (≥ 4 U) in this region. To choose this threshold for U-tract definition, we calculated the number of uridines in randomly sampled 8-nt tracts on both the forward (n=500) and reverse (n=500) strands of the *Syn* genome (n=1000 total tracts) (**Supplementary Fig. 1**). The cumulative distribution of uridine counts in the random 8-nt tracts was plotted. There was a 25% chance of finding ≥ 3 U, 8% chance of ≥ 4 U, and 2% chance of ≥ 5 U. Setting the threshold at ≥ 4 U thus reduced the risk of randomly identifying U-tracts while avoiding excessive stringency.

For the defined ends with U-tracts, we also calculated the maximum number of consecutive uridines in the U-tract. To be counted as having consecutive uridines, at least one ‘UU’ dinucleotide must be found in the 8-nt region. U-tracts with no consecutive uridines must still have 4 uridines to pass the threshold for U-tract definition so will take the form of either ‘UNUNUNUN’ or ‘NUNUNUNU’, where ‘N’ is any non-U ribonucleotide. Such cases (n=4) are omitted from **Fig. 2e**, which shows the remaining 221 U-tract defined ends.

*Sequence Logos.*

To determine the sequence features surrounding defined ends, we generated sequence logos for the 15 nt upstream of and including the peak position and the downstream 10 nt for defined ends with U-tracts or without U-tracts using WebLogo^2^. Note that WebLogo outputs DNA-based signatures, so T should be interpreted as a U in the RNA.

*Assessing RNA Secondary Structure at Defined Ends.*

We used Vienna RNAfold (Version 2.4.13)^3^ to calculate the MFE of 30-nt sliding sequence windows starting 70 nt upstream of and including the 3′ peak position to 40 nt downstream of the peak (81 total windows) for all defined ends with or without U-tracts. MFEs for all the windows were plotted against window end positions relative to the peak position, generating a trace that represents the level of RNA structure across the 110-nt sequence context of a given defined end. MFE traces for all defined ends in each U-tract category and species were overlaid to generate a spaghetti plot. Traces with an MFE < -10 kcal/mol for any of the windows ending from 10 nt upstream of the peak (-10 on x-axis) to the peak itself (0 on x-axis) were colored red to convey the frequency of ends with high RNA secondary structure in the region upstream of the peak where intrinsic terminator hairpins are anticipated to fold. Traces that did not meet this criterion in the specified region were colored gray to convey the frequency of ends that are not highly structured in the terminator hairpin region. The overlaid pink and light gray traces on each plot represent the average MFE trace for ends classified as highly structured (red traces) and not highly structured (gray traces), respectively.

Relaxing the MFE threshold to < -9 kcal/mol increased the proportion of structured U-tract defined ends in *Syn* to 77%. Still, 8% of *Syn* ends in this category consistently showed insufficient structure (MFE ≥ -5 kcal/mol) to correspond to a typical intrinsic terminator, compared to just 2% in *Bsu* and 3% in *Eco*.

*Over-Expression and Purification of Cyanobacterial Proteins.*

Expression and purification of cyRNAP, cy-rpoD1, cyNusG, and cyNusA were performed as follows. All expression plasmids were provided by Dr. Yulia Yuzenkova.

CyRNAP: The cyRNAP core was purified as reported previously^4,5^. In brief, the core enzyme of cyRNAP was overexpressed in *E. coli* BL21(DE3) cells (NEB, Catalog No. C2527H) transformed with a pET28a expression vector containing the genes encoding α, β, β′1, β′2, and ω subunits (β and β′2 contain a Strep-tag and His-tag, respectively). The cells were grown in LB media supplemented with kanamycin (50 μg/mL) at 37ºC to an OD_600_ of ~0.6 then induced with IPTG (1 mM) and grown overnight at 22ºC. Cells were harvested by centrifugation and resuspended in lysis buffer (50 mM Tris-HCl, pH 8.0, 250 mM NaCl, 10% glycerol, 1 mM β-mercaptoethanol, and cOmplete^TM^ EDTA-free protease inhibitor cocktail [Roche, Catalog No. 11873580001; used as per manufacturer’s instructions]). The suspension was lysed by sonication (40% amplitude, 5 s on/15 s off cycles, total on-time 5 min), and the lysate was clarified by centrifugation at 18,000 g at 4ºC. The supernatant was purified sequentially at 4ºC using a HisTrap HP column (5 mL; Cytiva, Catalog No. 17524801) followed by a StrepTrap XT column (1 mL; Cytiva, Catalog No. 29401317). For Ni-affinity purification, the cleared lysate was applied to the HisTrap column pre-equilibrated with Buffer I (50 mM Tris-HCl, pH 8.0, 250 mM NaCl, 2 mM β-mercaptoethanol, and 10% glycerol). The column was washed with Buffer I containing 30 mM imidazole and eluted with Buffer I containing 200 mM imidazole. Eluted fractions were pooled and diluted with 100 mM Tris-HCl, pH 8.0, to adjust the final NaCl concentration to 150 mM for subsequent purification. For Strep-tag affinity purification, the sample was applied to a StrepTrap XT column pre-equilibrated with Buffer W (100 mM Tris-HCl, pH 8.0, 150 mM NaCl, 1 mM EDTA), washed with 3-4 column volumes of Buffer W, and eluted with Buffer E (100 mM Tris-HCl, pH 8.0, 150 mM NaCl, 1 mM EDTA, and 2.5 mM desthiobiotin). The purified cyRNAP was analyzed by SDS-PAGE, concentrated using an Amicon Ultra centrifugal filter unit (100 kDa MWCO; Millipore, Catalog No. UFC910008), and buffer-exchanged into storage buffer (40 mM Tris-HCl, pH 8.0, 200 mM KCl, 1 mM EDTA, 1 mM DTT, and 5% glycerol). The enzyme was mixed with an equal volume of 100% glycerol, snap frozen in liquid nitrogen, and stored at -80ºC until use.

Cy-rpoD1: Recombinant rpoD1 was purified from E. coli BL21(DE3) cells carrying the plasmid pET28a-TEV-rpoD1 following a previously described protocol^4^ with minor modifications. Cells were grown in LB medium supplemented with kanamycin (50 µg/mL) at 37ºC until reaching an OD_600_ of ~0.6. Protein expression was induced with 0.4 mM IPTG, and the culture was incubated for 16 hr at 18ºC. The harvested cells were resuspended in lysis buffer (50 mM Tris-HCl, pH 7.9, 300 mM NaCl, 5% [v/v] glycerol, 2 mM β-mercaptoethanol, and protease inhibitor cocktail) and lysed by sonication. The clarified lysate was applied to a HisTrap HP column (1 mL; Cytiva, Catalog No. 29051021) pre-equilibrated with Buffer I (50 mM Tris-HCl, pH 7.9, 300 mM NaCl, 2 mM β-mercaptoethanol, and 5% glycerol) containing 10 mM imidazole. The column was washed with Buffer I containing 30 mM imidazole and eluted with Buffer I supplemented with 400 mM imidazole. The eluted fractions were treated with TEV protease during dialysis against buffer (20 mM Tris-HCl, pH 7.8, 300 mM NaCl, 5% [v/v] glycerol, and 2 mM β-mercaptoethanol) to remove the His-tag. The sample was then applied again to a HisTrap HP column (1 mL) pre-equilibrated with buffer I, and flow through was collected. The sample was diluted to reduce the NaCl concentration to 100 mM and then applied to a HiTrap Q HP anion exchange column (1 mL; Cytiva, Catalog No. 29051325) pre-equilibrated with 10% Buffer B (Buffer A: 20 mM Tris-HCl, pH 7.9, 0.2 mM EDTA, 1 mM DTT, and 5% glycerol; Buffer B: Buffer A with 1 M NaCl). The bound protein was eluted using a linear salt gradient between Buffer A and Buffer B. Fractions containing rpoD1 were pooled, concentrated using an Amicon Ultra-4 centrifugal filter unit (3 kDa MWCO; Merck Millipore, Catalog No. UFC800308), and buffer exchanged into storage buffer (20 mM Tris-HCl, pH 7.9, 200 mM NaCl, 5% glycerol, 1 mM DTT, and 0.2 mM EDTA). The purified protein was mixed with an equal volume of 100% glycerol, snap frozen, and stored at -80ºC until use.

CyNusG and CyNusA: For the preparation of cyanobacterial NusA and NusG, E. coli BL21(DE3) cells were transformed with the respective expression plasmids and grown in 500 mL LB medium at 37ºC to an OD_600_ of ~0.5-0.6. Protein expression was induced with 0.5 mM IPTG, and the cultures were incubated for 4 hr at 30ºC. Cell pellets were resuspended in 50 mL lysis buffer (40 mM Tris-HCl, pH 7.9, 300 mM NaCl, 2 mM β-mercaptoethanol, and 5% glycerol) supplemented with one tablet of cOmplete^TM^ EDTA-free protease inhibitor cocktail and 0.1 mg/mL lysozyme (Sigma-Aldrich, Catalog No. L6876). Cells were lysed by sonication as described above, and the lysate was clarified by centrifugation. The cleared lysate was applied to a HisTrap HP column (1 mL) pre-equilibrated with Buffer I (40 mM Tris-HCl, pH 7.9, 300 mM NaCl, 2 mM β-mercaptoethanol, and 5% glycerol). The column was washed with Buffer I containing 30 mM imidazole, and bound proteins were eluted with Buffer I supplemented with 250 mM imidazole. Fractions containing NusA or NusG were pooled and concentrated using Amicon Ultra centrifugal filters. NusA was subjected to an additional purification step prior to gel filtration. Namely, the salt concentration of the pooled NusA fractions was adjusted to 100 mM, and the sample was applied to a HiTrap Q HP anion exchange column (1 mL) pre-equilibrated with 10% Buffer B (Buffer A: 20 mM Tris-HCl, pH 7.9, 0.2 mM EDTA, 1 mM DTT, and 5% glycerol; Buffer B: Buffer A containing 1 M NaCl). NusA was eluted using a linear salt gradient between Buffer A and Buffer B. Fractions containing NusA were pooled and concentrated using Amicon filters. Both NusA and NusG were further purified by size-exclusion chromatography on a HiLoad 16/600 Superdex 75 pg column (Cytiva, Catalog No. 28989333) equilibrated with storage buffer (20 mM Tris-HCl, pH 7.9, 200 mM NaCl, 0.2 mM EDTA, 0.5 mM DTT, and 5% glycerol). Fractions containing pure NusA or NusG were pooled, concentrated, mixed with an equal volume of 100% glycerol, snap frozen in liquid nitrogen, and stored at -80ºC until use.

*DNA Templates for* In Vitro *Transcription Termination Assay.*

DNA templates for the multi-round transcription assay were synthesized as gene blocks (Integrated DNA Technologies) and PCR amplified using forward and reverse primer sets. Each DNA template contained a promoter region with canonical T7A1 motifs (–10/–35 elements), a transcription start site, and a terminator region comprising either a short (Term1, T-1849) or long (Term2, T-0252) stem-loop structure, followed by an additional short downstream sequence beyond the termination site. Sequences for the two tested terminator templates and amplification primers were: Synpcc_term1 (T-1849), 5′-CGGAATTCCGAAGA

TTAATTTAAAATTTATCAAAAAGAGTATTGACTTAAAGTCTAACCTATAGGATACTTACAGCCATATGAGAGTTGCTAGTCGCTTCCCTGTCCAGTCCTCCTGTCTGTCTTAAGCAGCTTAGGCGCGATCGCCTAGGCTGTTTTTTTGACCGTCCATTGCGTGATCGGTGCCAGCGATTTCCAGCCAAGCTTGGG-3′; Synpcc_term2 (T-0252), 5′-CGGAATTCCGAAGATTAATTTAAAATTTATCAAAAA

GAGTATTGACTTAAAGTCTAACCTATAGGATACTTACAGCCATATGAGAGTTGAAACCCGCCGATACAGAGGCGTACTATTGCGGCTAGAGTACCGCGGCTCAACCGATGCCAAGGTCTCTAGCTGTTTTTGTGCTTGGCTGCGGGAGCAATCCAGAAGCTGTGCGGCCAAGCTTGGG-3′; Synpcc_F, 5′-CGGAATTCGAAGACTCAGTTTAACATTTATC-3′; Synpcc_term1R, 5′-CCCAAGCTTGGCTGGA

AATCGCTG-3′; Synpcc_term2R, 5′-CCCAAGCTTGGCCGCACAGCTTC-3′.

In Vitro *Transcription Termination Assay.*

DNA templates for *in vitro* transcription were PCR amplified using gene blocks, as described above. DNA-RNAP complexes were assembled by incubating equal volumes of 2x DNA template (100 nM) and 2x reaction master mix for 5 min at 37ºC. The master mix contained 400 nM cyRNAP core enzyme, 800 nM rpoD1, 100 µg/mL bovine serum albumin, and 2x transcription buffer (1x = 40 mM Tris-HCl, pH 8.0, 5 mM MgCl_2_, 5% glycerol, 0.1 mM EDTA, and 4 mM dithiothreitol [DTT]). RNAP and rpoD1 were added from a 10x stock solution containing 2 µM RNAP and 4 µM rpoD1 in enzyme dilution buffer (20 mM Tris-HCl, pH 8.0, 40 mM KCl, 1 mM DTT, and 50% glycerol). To the preformed DNA-RNAP complexes (2 µL aliquots), 1 µL of 4x protein mix containing NusA and/or NusG (final concentrations of each, 1.0 µM) was added, followed by incubation for 5 min at room temperature. Transcription was initiated by adding 1 µL of 4x rNTP mix (final 1x = 20 mM KCl, 250 µM each of ATP, GTP, and CTP, 50 µM UTP, and 0.5 µCi [α-^32^P] UTP in 1x transcription buffer). Reactions were incubated for 15 min at 37ºC and terminated by adding an equal volume of 2x stop/loading buffer (40 mM Tris base, 20 mM Na_2_EDTA, 0.2% SDS, 0.05% bromophenol blue, and 0.05% xylene cyanol in formamide). RNA products were resolved on standard 5% polyacrylamide-urea sequencing gels, dried, and exposed using a phosphor screen. Signals were visualized using an Amersham Typhoon Phosphorimager, and band intensities were quantified using ImageJ to calculate the percent transcription termination (%T) as the ratio of terminated product to terminated and run-off product. The *in vitro* transcription termination experiment was performed twice, with each replicate derived from an independent reaction mixture. A representative gel is shown in **Supplementary Fig. 2**.

*TransTermHP Intrinsic Terminator Prediction for Defined and Diffuse Ends.*

To perform intrinsic terminator prediction, TransTermHP Version 2.07^6^ was applied to the *Syn* genome sequence without genome annotations and a confidence cutoff of 60 (-c 60). The prediction was performed separately on the forward and reverse strands of the genome sequence, and then the outputs from each run were combined for subsequent analysis.

Next, we determined the number of genes in each end category that had a predicted high-confidence intrinsic terminator in the downstream intergenic region. The intergenic region was defined as the sequence between the stop codon of the gene under consideration and the start codon of the next gene on the same strand. To stay within a biologically relevant range, terminator searches were limited to the first 1 kb if the intergenic region exceeded that length. If multiple intrinsic terminators were detected in the search region, we retained the terminator with the highest confidence score. After identifying genes with a predicted intrinsic terminator in the search region, we determined the number of genes in each end category with a high-confidence terminator by filtering for those with a confidence score > 80. Our cutoff is slightly more stringent than that used to define TransTermHP high-confidence terminators (confidence score ≥ 76)^6^.

*Mapping Transcript Tapering at Diffuse Ends.*

For diffuse ends, our pipeline identifies the position where read density drops at least ~70% (3.5-fold) relative to the region upstream of the stop codon and records this as the diffuse end position. To estimate the tapering length of mapped reads as they ramp down at diffuse transcript termini, we measured the distance from the stop codon to this endpoint (“post-stop taper length”) in **Fig. 4a**. The read counts from WT diffuse ends were also used to generate the diffuse end traces in **Fig. 4b**. For each gene, the read density was calculated in 100 bp windows spaced 15 bp apart, starting 513 bp upstream of the center of the 75 bp window where the density drop was identified and extending 262 bp downstream. The read densities for each window were then normalized to the read density in the gene body (calculated across a window extending 30-105 bp upstream of the stop codon). Diffuse ends with multiple 3′ peaks (diffuse peaks, n=75) were excluded from this analysis. A randomly selected subset of 10 diffuse ends is shown in the main text; all analyzed diffuse ends can be seen in **Supplementary Fig. 3**.

*Sequence Bias Analysis at TU Ends and TU-Internal Gene Ends.*

To analyze the sequence composition around *Syn* defined and diffuse ends, the fractions of A, T, C, and G nucleotides were determined in windows extending 51 or 201 bp upstream and downstream of (1) the 3′ peak for all the identified defined ends (n=388), (2) the downstream end of the window corresponding to the density drop for diffuse ends (excluding diffuse peaks) (n=274), and (3) the downstream end of the final window reached by the end calling pipeline for all *Syn* genes in TUs (i.e., TU-internal genes) (n=544). Statistical comparisons were performed using the unpaired two-sided t-test (scipy.stats.ttest_ind) with unequal variance assumed (equal_var=False).

Synechocystis *RNase J Depletion Analysis.*

To compare the TU end positions in RNA-seq data from WT and RNase J-depleted *Synechocystis* sp. PCC 6803^7^, the end calling pipeline (see above section) was modified to eliminate use of 5′ and 3′ peak information. Briefly, the modified pipeline was used to scan for a drop in read density exceeding 4-fold between the gene body (30-105 bp upstream of the stop codon) and a sliding 75-bp window that could extend as far as the stop codon of the following gene on the same strand. The end position of the window where the read density first dropped below 4-fold was recorded. Classification was performed using the gene annotations corresponding to NC_000911.1, excluding genes shorter than 155 bp or with fewer than 5 reads/bp across the gene body (excluding 45 bp on either end).

To determine if TU ends tended to shift downstream in the RNase J depletion strain, we calculated the positional offset between the TU end identified in WT and RNase J-depleted *Synechocystis* (Position_depletion_ – Position_WT_). Positive offsets indicate that the drop in density was observed farther downstream in the depletion dataset. The data shown in **Fig. 5** are limited to genes that were identified as terminated in the WT data and exceeded 100 reads/bp in the gene body.

*BLAST Searches for Termination Factor Homologs in Cyanobacteria.*

To test for cyanobacterial homologs of known archaeal, eukaryotic, and organellar termination factors, the following BLAST searches were performed: (1) a protein-protein BLAST (blastp) of *A. thaliana* RHON1 (GenBank: OAP15819.1) against the *S. elongatus* PCC 7942 (taxid:1140) non-redundant protein sequences (nr) database, and an expanded blastp search against the entire cyanobacterial phylum (taxid:1117) nr database; (2) blastp of MTERF1 from *Homo sapiens* (UniProtKB/Swiss-Prot: Q99551.1) and *Mus musculus* (isoform X1, NCBI RefSeq: XP_017176489.1) against the cyanobacterial (taxid:1117) and *S. elongatus* PCC 7942 (taxid:1140) nr databases, and (3) blastp and Domain Enhanced Lookup Time Accelerated BLAST (DELTA-BLAST) of FttA from a representative hyperthermophilic archaeon *Pyrococcus abyssi* GE5^8^ (UniProtKB/Swiss-Prot: Q9V0P0.2) against the *S. elongatus* PCC 7942 (taxid:1140) nr database.

*Displacement and Readthrough Fold-Change Calculations for* mfd *Knockout.*

To assess the impact of *mfd* knockout on TU end positions, we calculated the positional offset of TU ends in each end class between WT and the Δ*mfd* strain (Position_Δ_*_mfd_* – Position_WT_). Biological reproducibility between Δ*mfd* replicates was similarly assessed by computing the offset between replicates (Position_Δ_*_mfd_* _rep 2_ – Position_Δ_*_mfd_* _rep 1_). TU end positions were defined as either the 3′ peak position (for defined ends) or the end of the downstream window where the density drop threshold is met (for diffuse ends), as determined by the end calling pipeline.

To quantify the effect of *mfd* knockout on transcriptional readthrough (RT) at defined ends, we used the following log_2_-based ratios:

log_2_($\frac{WT RT}{\Delta mfd\mathrm{RT}}$)

to compare readthrough levels between WT and Δ*mfd* replicates, where negative values on the x-axis indicate increased readthrough in the Δ*mfd* strain, and

log_2_($\frac{\Delta mfd rep 1 RT}{\Delta mfd rep 2 RT}$)

to evaluate biological reproducibility between knockout replicates. RT values were calculated using the end calling pipeline.

*Analysis of Photosystem Genes.*

40 genes encoding components of the photosystems were identified from the gene descriptions provided in Rubin *et al*.^9^; 17 of these genes corresponded to TUs with end types assigned here (13 defined ends, 2 diffuse ends, and 2 unclear). A Fischer’s exact test was used to assess the significance of the difference between the frequency of defined ends among these photosystem TUs vs. all classified TUs (76% vs. 52%, n = 17 photosystem vs. 751 total).

*COG Functional Analysis.*

Clusters of Orthologous Genes (COG) ID and functional category assignments for the *Syn* chromosome^10^ were downloaded in January 2026. If multiple COG functional categories were assigned to a given COG footprint, the primary category was taken. The end type (defined or diffuse) corresponding to each COG footprint was assigned using the associated gene ID. The primary COG functional categories associated with each gene are listed in **Supplementary Table 1**. Note that a given gene may have multiple assignments if there were multiple COG footprints identified in the gene body.

To identify functional categories with differential representation among genes with a given end type (defined or diffuse), the frequency of each COG functional category was determined across all COG footprints with that end type and compared to the background frequency of the functional category across the entire *Syn* chromosome. Frequencies at least 2-fold higher or lower than the background were considered to exhibit differential representation, and the statistical significance was assessed using a Fischer's exact test with a Bonferroni-corrected p-value of 0.002. None of the functional categories exhibited significant differential representation among genes with defined or diffuse ends.

The same process was used to assess functional categories among genes downstream of a given end type (on the same strand). Again, no significant differences in frequency were identified for any of the functional categories.

**References**

1. Lalanne, J.-B. *et al.* Evolutionary convergence of pathway-specific enzyme expression stoichiometry. *Cell* **173**, 749-761.e38 (2018).

2. Crooks, G. E., Hon, G., Chandonia, J.-M. & Brenner, S. E. WebLogo: a sequence logo generator. *Genome Res.* **14**, 1188–1190 (2004).

3. Lorenz, R. *et al.* ViennaRNA Package 2.0. *Algorithms Mol. Biol.* **6**, 26 (2011).

4. Shen, L. *et al.* An SI3-σ arch stabilizes cyanobacteria transcription initiation complex. *Proc. Natl. Acad. Sci.* **120**, e2219290120 (2023).

5. Qayyum, M. Z. *et al.* Structure and function of the Si3 insertion integrated into the trigger loop/helix of cyanobacterial RNA polymerase. *Proc. Natl. Acad. Sci.* **121**, e2311480121 (2024).

6. Kingsford, C. L., Ayanbule, K. & Salzberg, S. L. Rapid, accurate, computational discovery of Rho-independent transcription terminators illuminates their relationship to DNA uptake. *Genome Biol.* **8**, R22 (2007).

7. Cavaiuolo, M., Chagneau, C., Laalami, S. & Putzer, H. Impact of RNase E and RNase J on global mRNA metabolism in the cyanobacterium *Synechocystis* PCC6803. *Front. Microbiol.* **11**, 1055 (2020).

8. Erauso, G. *et al.* *Pyrococcus abyssi* sp. nov., a new hyperthermophilic archaeon isolated from a deep-sea hydrothermal vent. *Arch. Microbiol.* **160**, 338–349 (1993).

9. Rubin, B. E. *et al.* The essential gene set of a photosynthetic organism. *Proc. Natl. Acad. Sci.* **112**, E6634–E6643 (2015).

10. Galperin, M. Y. *et al.* COG database update 2024. *Nucleic Acids Res.* **53**, D356–D363 (2025).
